# Ultra‐Sensitive Dual‐Resonator Graphene Pressure Sensor with Temperature Self‐Compensation

**DOI:** 10.1002/advs.202517536

**Published:** 2025-11-05

**Authors:** Zhen Wan, Cheng Li, Zhengwei Wu, Pengcheng Zhao, Yang Liu, Wanting Li, Shangchun Fan, Wei Jin

**Affiliations:** ^1^ School of Instrumentation Science and Opto‐Electronics Engineering Beihang University Beijing 100191 China; ^2^ Research Institute of Beihang University in Shenzhen Shenzhen 518055 China; ^3^ Aerospace Information Research Institute Chinese Academy of Sciences Beijing 100190 China; ^4^ Leibniz Institute of Photonic Technology 07745 Jena Germany; ^5^ Department of Electrical and Electronic Engineering The Hong Kong Polytechnic University Hong Kong Kowloon 999077 China

**Keywords:** dual‐resonator, graphene pressure sensor, temperature self‐compensation, ultra‐sensitive

## Abstract

Silicon resonator sensors have limitations in detecting small pressure changes due to their structural dimensions. Graphene nanomechanical resonators, with their ultra‐small thickness and excellent mechanical properties, offer the opportunity to break this limitation. Here, a highly sensitive graphene nanomechanical pressure sensor with integrated temperature self‐compensation is reported. It consists of two vacuum anode‐bonded graphene resonators: one is sensitive to pressure and temperature while the other to temperature only, allowing for the cancellation of thermal effects via detecting the difference in the resonant frequencies. A sensitivity of 24.1 kHz kPa^−1^ is achieved over the pressure range of 0.001 to 500 kPa, 68 times higher than the state‐of‐the‐art silicon pressure sensors. The full‐scale (FS) hysteresis error is 0.31% with a repeatability of 0.75% in three forward and reverse stroke pressure tests. Within the temperature range of −40 to 120 °C and the pressure range of 0.001 to 500 kPa, the maximum pressure error is 6.51 kPa, giving an accuracy of 1.302% FS. The high performance of the device makes it promising for applications in aerospace, automotive, and healthcare industries as well as other fields requiring high‐sensitivity pressure measurements.

## Graphical abstract

This work presents a graphene resonator sensor based on a dual‐cavity interferometric scheme that achieves high sensitivity to pressure variations. Its lightweight and compact design enables drone‐based field experiments, underscoring the promise of graphene optomechanical devices for portable and scalable sensing applications.

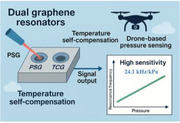

## 1 Introduction

Highly sensitive micro‐electromechanical system (MEMS) pressure sensors, capable of operating stably at atmospheric pressure, have garnered considerable attention from the aerospace to automotive industries.^[^ ^1^, ^2^, ^3^, ^4^ ^]^ Pressure applied to the sensing diaphragm induces strain or deflection, which is detectable through optical or electrical means.^[^ ^5^ ^]^ Resonant pressure sensors, which detect the change of resonant frequency with pressure, offer notable advantages, including high sensitivity, stability, and digital output.^[^ ^6^ ^]^ Recently, nano‐electromechanical systems (NEMS) utilizing 2D materials have been explored for pressure sensing.^[^ ^7^ ^]^ Resonators fabricated with 2D materials have demonstrated high sensitivity for the measurement of pressure,^[^ ^8^, ^9^ ^]^ mass,^[^ ^10^ ^]^ acceleration,^[^ ^11^ ^]^ and thermal radiation.^[^ ^12^ ^]^ Among many 2D materials, graphene stands out as an ultrasensitive membrane for pressure detection, owing to its atomic‐level thickness, high Young's modulus, exceptional fatigue property, and strong adhesion.^[^ ^13^, ^14^, ^15^ ^]^ The integration of graphene into conventional MEMS pressure sensors has significantly enhanced the responsiveness and miniaturization of these sensors.^[^ ^16^ ^]^

Theoretical modelling of graphene resonant pressure sensors utilizing a squeezed membrane shows that the sensitivity of resonant frequency to pressure can be much greater than that of a conventional silicon resonator.^[^ ^17^ ^]^ However, experimental results deviate considerably from the theory, primarily due to the slippage effect at the graphene membrane‐substrate interface.^[^ ^18^ ^]^ It has been demonstrated that depositing SiO_2_ on the edge of the graphene membrane enhances the van der Waals interaction at the graphene‐substrate interface, thereby improving pressure sensitivity.^[^ ^19^ ^]^ We have previously reported on a graphene micro‐optical‐electro‐mechanical system (MOEMS) resonant pressure sensor, wherein the graphene membrane is encapsulated within a vacuum chamber through an anodic bonding process, effectively reducing energy loss due to air damping of the membrane vibration.^[^ ^20^ ^]^ Nonetheless, temperature fluctuation of the environment exerts a non‐negligible influence on the sensor output, posting a critical challenge that must be effectively addressed.

In this work, we report a highly sensitive, temperature self‐compensated graphene resonant pressure sensor with overall dimensions of 1 cm × 1 cm × 0.4 cm. Unlike approaches that employ two independent sensors with subsequent temperature compensation, the device co‐integrates a pressure‐sensitive graphene (PSG) resonator on a thin diaphragm and a temperature‐compensating graphene (TCG) resonator on the thick substrate within the same vacuum‐bonded package and optical readout. This co‐packaging yields closely matched temperature coefficients and a shared photothermal environment; temperature‐related drifts become common‐mode and are removed by a differential‐frequency readout that preserves only the pressure contribution. The sensor attains a pressure sensitivity of 24.1 kHz kPa^−1^, which is two orders of magnitude higher than typical silicon‐based resonant pressure sensors.^[^ ^21^, ^22^, ^23^, ^24^ ^]^ The sensor demonstrated a maximum pressure error of 6.51 kPa across operational conditions of −40 to 120 °C and 1 Pa to 500 kPa, achieving an accuracy of 1.302%FS. The maximum time drift is 0.014% over 4800 s and 0.160% over a duration of 30 days. This exceptional stability is attributed to the vacuum anode‐bonded encapsulation structure of the sensor coupled with the SiO_2_ edge deposition on the graphene resonator, which mitigates the effect of unstable pressure in the vacuum chamber and enables the sensor to detect a minimum pressure of 8.64 Pa. We have successfully mounted the sensor on an unmanned aerial vehicle (UAV) to demonstrate the sensor's potential application in altimetry, and have achieved a remarkable height accuracy of better than ±1 m.

## 2 Results

### 2.1 Sensing Structure and Mechanism

**Figure 1:**
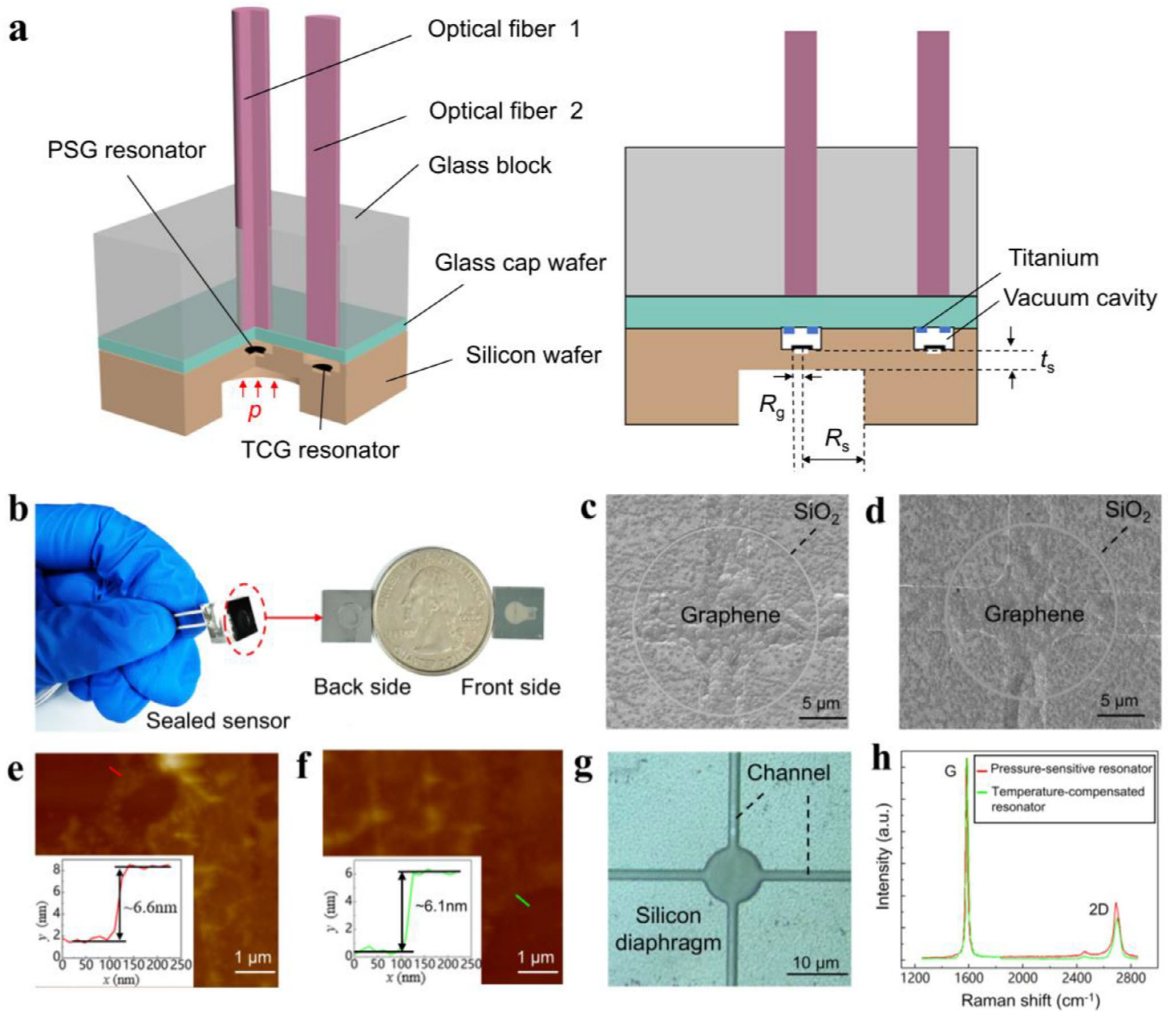
The graphene resonant pressure sensor. a) Schematic of the pressure sensor. *p* is the external pressure; *t*_s_ is the thickness of the silicon diaphragm; *R*_s_ and *R*_g_ are the radius of the silicon diaphragm and graphene membrane, respectively. b) Photograph of a fabricated sensor. The image of a quarter dollar is included to show the size of the sensor. c) SEM image of the PSG resonator. SiO_2_ is deposited on top of graphene near the edge of the circular pit to enhance the van der Waals interaction at the graphene‐substrate interface. d) SEM image of the TCG resonator. The minor image differences primarily originate from the supporting layers and routine transfer/imaging conditions, both resonators use the same material stack and edge‐clamping structure. e) AFM characterization and height profile of the PSG resonator. f) AFM characterization and height profile of the TCG resonator. g) Air channels with a width of ≈1 µm etched at the edges of circular pits above the silicon diaphragm/substrate. h) Raman spectra of the PSG and TCG resonators.

As illustrated in **Figure** **1**a, the pressure sensor is composed of a silicon diaphragm fabricated on a silicon wafer substrate, two suspended graphene resonators, a glass cap wafer, and a glass block with two hollow channels to house two single‐mode optical fibers (SMFs). Specifically, the two graphene resonators, i.e., the PSG resonator and the TCG resonator, are located within two circular holes made on the silicon wafer separated by a distance of 3.5 mm. The PSG resonator, which is sensitive to both pressure and temperature, is situated above a small pit at the bottom of the hole on top of the thin silicon diaphragm. In contrast, the TCG resonator is located above the pit at the bottom of the second hole on the thick silicon substrate, rendering it insensitive to pressure but remaining responsive to temperature. The PSG and TCG resonators were encapsulated within the two holes or chambers between the silicon wafer and the glass cap wafer via vacuum anodic bonding. During the annealing and anodic bonding processes, it was noted that the gas trapped beneath the graphene membranes could not be released rapidly, resulting in uneven pressure distribution between the top and bottom surfaces of the graphene membrane. This pressure imbalance leads to damage of the graphene resonators. To mitigate this effect, we fabricated four narrow air channels on a silicon diaphragm/substrate, as shown in Figure 1g, to ensure the integrity of the graphene membranes. This configuration facilitates more accurate pressure measurement by detecting the differential resonant frequency of the two graphene resonators.

When a sinusoidally modulated laser beam (excitation light) irradiates the graphene resonator, the photothermal effect converts periodic optical energy into thermal strain, thereby driving mechanical vibration of the graphene membrane. By adjusting the laser modulation frequency to match the resonant frequency of the resonator, maximum vibrational amplitude is achieved. The vibration of the graphene membrane is detected via a Fabry–Pérot (F–P) interferometric cavity formed between the graphene membrane and the fiber end face, which converts the vibration into a light intensity variation of a second laser beam (detection light) at the output of the F–P cavity. The resonant frequency is determined by monitoring the change in the reflected light intensity as a function of the modulation frequency of the excitation beam. The silicon diaphragm deforms under the applied pressure, which alters the tension on the graphene membrane, leading to a shift in its resonant frequency. The relationship between the first‐order resonant frequency *f*_p,_ and the applied pressure *p* may be expressed as^[^ ^20^ ^]^

(1) $$f_{p}=\frac{2.404}{2\pi R_{g}}\sqrt{\frac{1}{\rho_{g}}\left(S_{0}+\frac{E_{g}t_{g}}{\left(1-\mu_{g}\right)}\frac{3p\left(1-{\mu_{s}}^{2}\right){R_{s}}^{2}}{8E_{s}{t_{s}}^{2}}\right)}$$

where *S*_0_, *E*_g_, *µg*, *t*_g_, *ρ*_g,_ and *R*_g_ are the initial tension, Young's modulus, Poisson's ratio, thickness, areal mass density, and radius of the graphene membrane, respectively.^25^, ^26^, ^27^ *E*_s_, *µs*, *t*_s,_ and *R*_s_ are the Young's modulus, Poisson's ratio, thickness, and radius of the silicon diaphragm, respectively (see Note S1 and Figure S1, Supporting Information). The small radius and low mass of the graphene membrane per unit area contribute to the ultra‐sensitive pressure detection.^[^ ^28^, ^29^, ^30^ ^]^ In the meantime, the radius and thickness of the silicon diaphragm also affect the resonant frequency and sensitivity of the sensor.

The resonant frequencies of the two graphene resonators are recorded simultaneously to facilitate temperature compensation of the sensor. The PSG resonator and the TCG resonator have near‐identical thicknesses and diameters, which ensures approximately the same temperature sensitivities (refer to Note S2, Supporting Information). Consequently, the applied pressure *p* can be determined by using^[^ ^31^ ^]^

(2) $$p=\frac{s_{2}\left(f_{r1}-\bar{f_{01}}\right)-s_{1}\left(f_{r2}-\bar{f_{02}}\right)}{k_{1}s_{2}-k_{2}s_{1}}$$

where *f*_r1_ and *f*_r2_, $\bar{f_{01}}$_,_ and $\bar{f_{02}}$, *k*_1_ and *k*_2_, and *s*_1_ and *s*_2_ are the measured resonant frequencies, initial resonant frequencies, pressure sensitivities, and temperature sensitivities of the PSG and TCG resonators, respectively.

The two graphene resonators are encapsulated within two small chambers between a silicon wafer and a glass cap wafer through vacuum anode bonding (details see Note S3, Figures S2 and S3, Supporting Information). The fabricated structure is integrated with optical fibers for the transmission of the excitation and detection light beams. Two SMFs are securely fixed within the hollow channels on the glass block and aligned with the PSG resonator and the TCG resonator, respectively, utilizing a micro‐imaging system (details see Note S4 and Figure S4, Supporting Information). This configuration creates a low‐finesse F–P cavity between the fiber end face and the graphene resonator, and the reflected detection laser beam from the cavity is guided back via the SMF for demodulation. The photos of the encapsulated graphene pressure sensor are shown in Figure 1b, with an overall dimension of 1 cm × 1 cm × 0.4 cm (length × width × height). The F–P cavity lengths of the PSG and TCG resonators are 287 and 282 µm, respectively (details see Note S5 and Figure S5, Supporting Information).

### 2.2 Characterization of the Graphene Membranes

The surfaces of the PSG and TCG resonators were examined using scanning electron microscopy (SEM), revealing a complete graphene membrane with deposited SiO_2_ rings, as shown in Figure 1c,d. The deposition of SiO_2_ effectively inhibits edge vibrational modes, thereby improving the quality (*Q*) factor of the resonators.^[^ ^26^ ^]^ The thickness of the graphene membranes was measured by atomic force microscopy (AFM), yielding ≈6.6 nm for the PSG resonator and 6.1 nm for the TCG resonator (see Figure 1e,f). The small thickness difference of 0.5 nm between the two graphene resonators indicates a closely matched temperature sensitivity. To mitigate damage from excess membrane stresses during the annealing of the graphene resonator and the anode bonding of the sensor, four air channels, each 1 µm in width, were etched along the edges of the circular pits, as illustrated in Figure 1g. This design allows for the timely escape of gas trapped between the graphene and the substrate. The Raman spectra of the PSG and TCG resonator, presented in Figure 1h, exhibit the absence of the D peak at 1350 cm^−1^, indicating a low concentration of defects in the graphene and high‐quality graphene membrane transferred onto the silicon substrate.

### 2.3 Experimental Setup for Pressure Measurement

**Figure 2:**
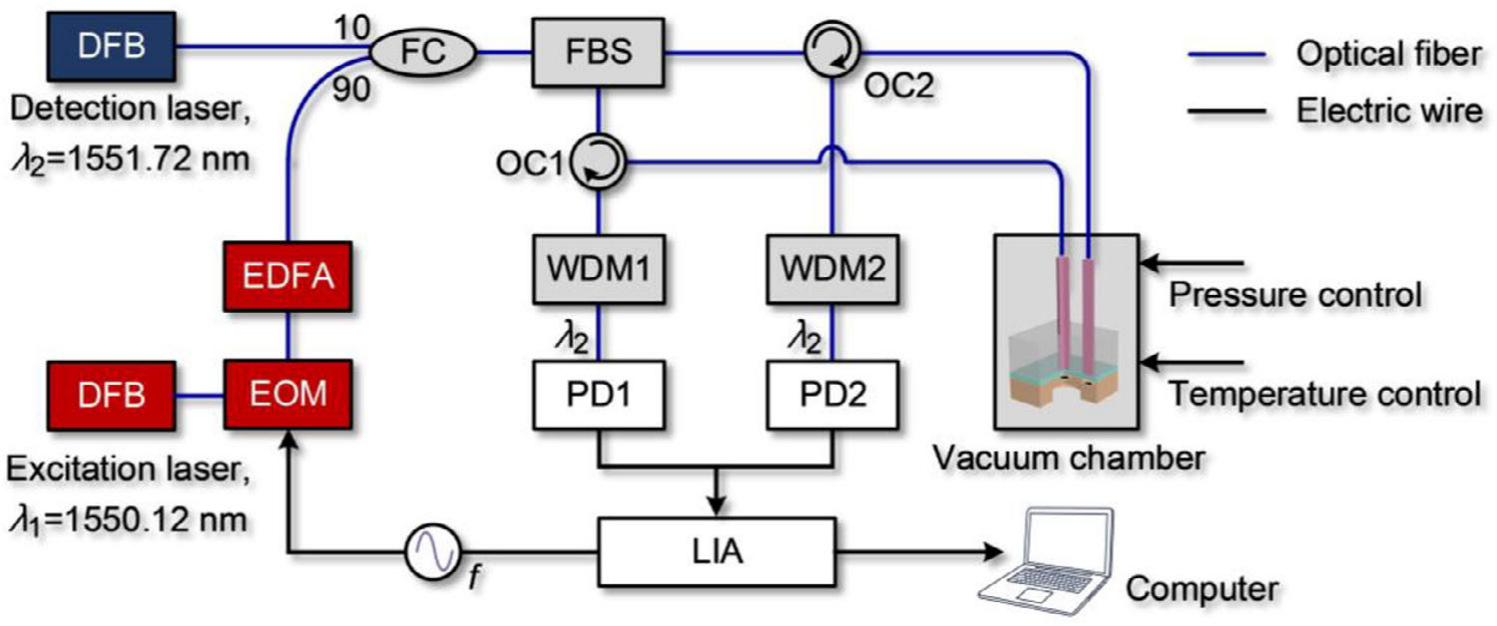
Schematic of the experimental system. The pressure sensor is placed inside a test chamber with controllable pressure and temperature.

The experimental setup for pressure sensing is illustrated in **Figure** **2**. Two distributed feedback (DFB) lasers with nominal wavelengths of *λ*_1_ = 1551.72 nm and *λ*_2_ = 1550.12 nm are used for excitation and detection. The excitation light induces vibrations in the graphene membranes through the photothermal effect, while the detection light monitors the vibrations from which the resonant frequencies of the membranes can be determined. The excitation light is sinusoidally modulated by an electro‐optical modulator (EOM) and then amplified by an Erbium‐doped fiber amplifier (EDFA). The modulated excitation light is combined with the detection light using an optical fiber coupler and then split into two beams by an optical fiber beam splitter (FBS). The FBS divides the light into two beams and transmits them separately to two optical circulators (OC). Each beam is directed into a graphene resonator through an optical circulator. The two reflected detection signals are collected by photodetectors (PD1 and PD2) after passing through wavelength division multiplexers (WDM1 and WDM2), which are used to filter out the excitation light. The signals from the PDs are then demodulated using a lock‐in amplifier (LIA) to determine resonant frequencies.

To avoid laser‐induced damage to the graphene resonators, the input optical signal is limited to less than 7 mW. Here, the excitation optical power was set to 0.8 mW and the detection optical power to 4 mW. It should be noted that, although measurements were conducted at room temperature, local laser heating can modify the membrane's material parameters and in‐plane stress, particularly when the device enters the nonlinear regime. To avoid this, the excitation and detection light beams were kept at low power, and the motion of graphene resonators were probed within the linear response regime. During experiments, the pressure sensor was placed inside a vacuum chamber with controlled pressure and temperature, which enables precise regulation of the sensor's operational environment.

### 2.4 Responsivity of the Pressure Sensor

**Figure 3:**
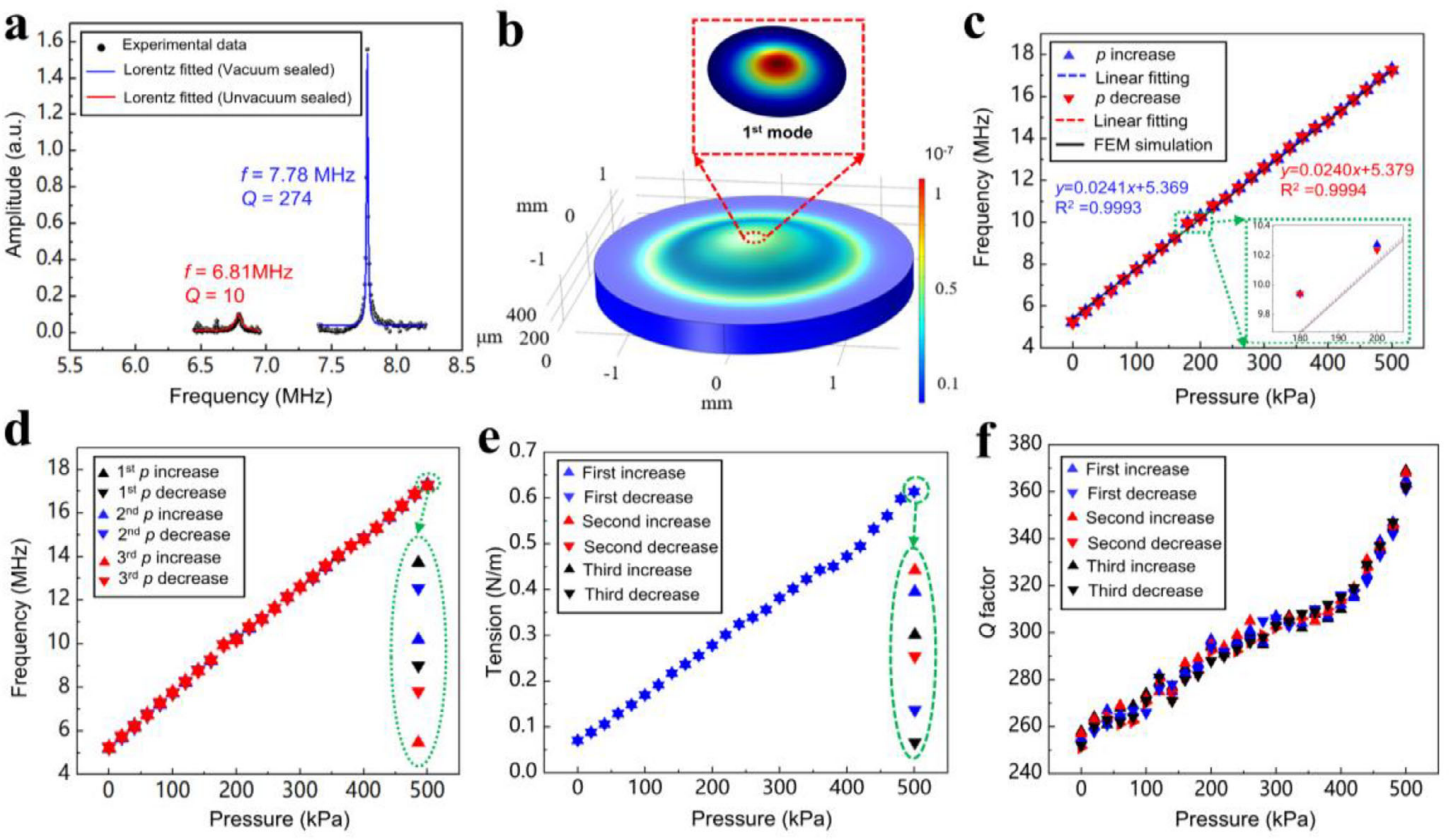
Performance of the PSG resonator. a) Measured amplitude‐frequency response of the PSG resonator before (red solid line) and after (blue solid line) vacuum‐sealing at the ambient pressure of 100 kPa. b) The finite element model (FEM) results of strain in the silicon diaphragm at 100 kPa. The red dashed box shows the fundamental vibration mode (1^st^ mode) of the PSG resonator. c) Measured resonant frequency in response to pressure. The blue and red triangles indicate the resonant frequency for increasing and decreasing pressure, respectively. The blue and red dashed lines indicate the results of linear fitting, while the black solid line represents the FEM simulation results. d) Repeatability for three forward and backward strokes. The different colored symbols represent individual strokes. e) Measured tension of the PSG resonator during three forward and backward pressure strokes. f) Measured the *Q* factor of the graphene resonator for three forward and backward strokes of pressure.

To mitigate the effects of air damping on the *Q* factor of the pressure sensor, vacuum anode bonding was employed for sensor encapsulation. The resonant characteristics of the sensor at room temperature were assessed before and after encapsulation, and the results are shown in **Figure** **3**a. The *Q* factor was determined from the full width at half maximum of the Lorentzian line for each resonance. Before encapsulation, the measured resonant frequency of the graphene resonator was 6.81 mHz, corresponding to a *Q* factor of 10 at 100 kPa and 25 °C. In contrast, the vacuum‐sealed graphene resonator exhibited a resonance frequency of 7.78 mHz and a significantly higher *Q* factor of 274. This represents a 27‐fold increase in the *Q* factor compared to the pre‐encapsulation state, primarily attributable to the reduced energy loss resulting from diminished air viscosity in the vacuum environment. Additionally, the SiO_2_ deposition along the edges effectively suppressed the edge vibration modes of the graphene resonator, thereby concentrating the vibrational energy in the center of the membrane and further enhancing the *Q* factor.^[^ ^19^, ^26^ ^]^

To further assess the experimental results, we conducted a finite element model (FEM) simulation using COMSOL Multiphysics (see Table S1, Supporting Information). As shown in Figure 3b, the maximum stress is at the center of the silicon diaphragm and gradually decreases toward the edge. Additionally, the subplot depicts the fundamental vibration mode of the graphene resonator. For the simulation analysis, we extracted the initial tension *S*_0_ of the graphene resonator based on the experimental data. Specifically, when the resonant frequency *f*_p_ = 5.24 mHz is at its minimum (Δ*p* = 0), this scenario corresponds to the absence of deformation in the silicon diaphragm and graphene membrane. Consequently, the initial tension *S*_0_ of the pressure‐sensitive resonators was calculated to be 0.07 N m^−1^. With this pre‐tension, we carried out numerical simulations to investigate the pressure response of the circular graphene membrane, leading to FEM simulation results of Figure 3c. It should be stated that, considering the edge clamping effect of SiO_2_, the influences of graphene membrane slipping and squeezed membrane damping on the resonant frequency are negligible and thus were excluded from the simulation.^[^ ^32^, ^33^ ^]^

The resonant frequencies obtained from the experiments and finite element simulations exhibit a high degree of consistency at 25 °C and the range of 0.001 to 500 kPa, both showing a positive correlation with pressure, as illustrated in Figure 3c. The coefficients of determination for the forward and reverse strokes are 0.9993 and 0.9994, respectively. Furthermore, it is noteworthy that the maximum deviation between the forward and backward fitted curves is 37.4 kHz, indicating that the maximum full‐scale hysteresis error of the PSG resonator is only 0.31%. The initial measured resonant frequency of the PSG resonator at a pressure of 0.001 kPa is 5.24 mHz, while this frequency increases to 17.27 mHz when the pressure reaches a maximum of 500 kPa. Across the pressure range of 0.001 to 500 kPa, the average responsivity of the pressure sensor is 24.1 kHz kPa^−1^. In comparison to the previously reported state‐of‐the‐art fully encapsulated silicon resonant pressure sensor, which exhibited a pressure sensitivity of 0.353 kHz kPa^−21^, the sensitivity of the fabricated graphene resonant pressure sensor is 68 times greater. However, it should be noted that the pressure sensor's response is directly influenced by the deformation of the silicon diaphragm, leading to non‐linearity at pressures exceeding 500 kPa. Figure 3d presents the pressure experimental results from three forward and reverse strokes, with a maximum standard deviation of 30.2 kHz, leading to a calculated repeatability of 0.75% (detailed calculations can be found in Note S6, Supporting Information).

During the cyclic pressure experiments, the deformation of the silicon diaphragm induced a corresponding deformation in the PSG resonator situated on its upper surface. This interaction resulted in an increase in the tension of the graphene membrane as pressure was applied, as shown in Figure 3e. This aligns with the expected behavior of the sensing structure.^[^ ^20^ ^]^ Figure 3f shows that the variation in the *Q* factor of the PSG resonator across the three pressure strokes is positively correlated with the applied pressure. The *Q* factor reached its maximum value of 369 at 500 kPa, significantly exceeding the *Q* factor of the graphene resonator prior to encapsulation. On one hand, the PSG resonator is encapsulated in a vacuum environment, which isolates the effect of viscous damping from air.^[^ ^19^, ^34^ ^]^ On the other hand, the deformation of the PSG resonator increases with pressure, leading to an increase in its surface tension, which suppresses the energy loss due to edge modes.^[^ ^35^, ^36^ ^]^

### 2.5 Stability of the Pressure Sensor

**Figure 4:**
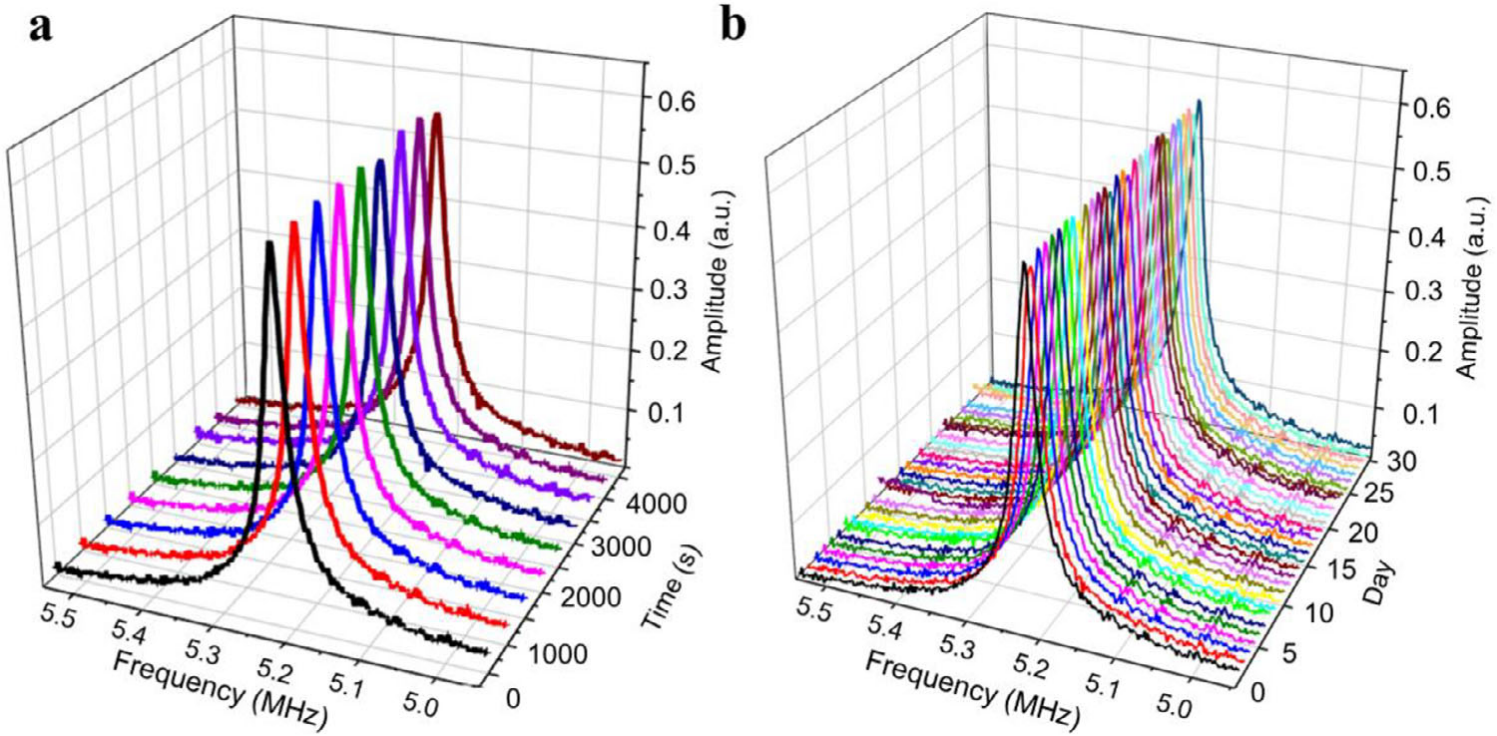
Stability of the graphene resonant pressure sensor. a) Resonant frequency vs time at the pressure of 0.001 kPa and the temperature of 25 °C. The experimental data are recorded from 0 to 4800 s in steps of 300 s. b) Resonant frequency vs day at the pressure of 0.001 kPa and the temperature of 25 °C. The experimental data were recorded daily for 30 days after the sensor signal output is stabilized.

To evaluate the stability of the PSG resonator, we conducted short‐term stability tests over a period of 4800 s and long‐term stability tests over 30 days. During the short‐term experiment, the pressure sensor was subjected to the pressure of 0.001 kPa and the temperature of 25 °C, and the results are shown in **Figure** **4**a. The maximum drift in resonant frequency was 0.74 kHz, corresponding to a short‐term time drift of 0.014% relative to the resonant frequency of 5.24 mHz. This performance exceeds the previously reported graphene resonant pressure sensors, which exhibited a drift of 0.055%.^[^ ^20^ ^]^ In the long‐term stability assessment, the maximum resonant frequency drift recorded was 8.43 kHz, corresponding to a long‐term time drift of 0.160% relative to the resonant frequency of 5.24 mHz, as shown in Figure 4b. This result also surpasses the long‐term stability of state‐of‐the‐art graphene resonant pressure sensors, which report a time‐drift of 0.55%.^[^ ^20^ ^]^

### 2.6 Temperature Compensation

The pressure and temperature sensitivity of the PSG and TCG in the sensor were studied in the temperature range of −40 to 120 °C and pressure range of 0.001 to 500 kPa. The measurements were conducted under an excitation laser power of 0.8 mW and a detection laser power of 4 mW. Both the PSG and TCG resonators exhibited a linear increase in the resonant frequencies with rising temperature.

**Figure 5:**
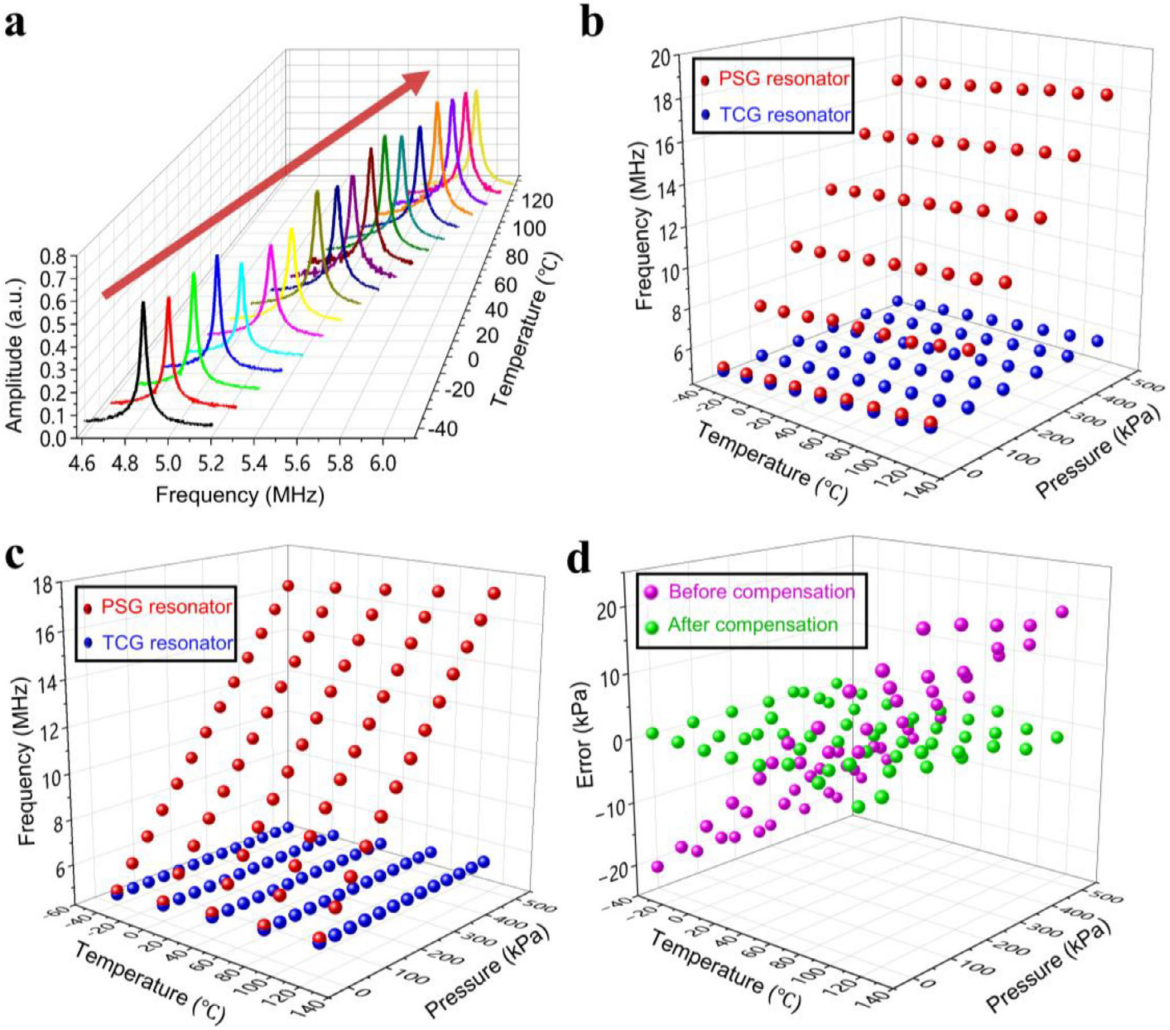
Temperature compensation of the graphene resonant pressure sensor. a) Variation of PSG resonant frequency with temperature at 0.001 kPa. b) Resonant frequency change of the PSG and TCG resonator with pressure (Maintaining constant temperature). c) Resonant frequency change of the PSG and TCG resonator with temperature (Maintaining constant pressure). d) Measurement error of the sensor before and after temperature compensation over the temperature range from −40 to 120 °C and the pressure range from 0.001 to 500 kPa.

**Figure** **5**a shows a shift in resonance for the PSG at 0.001 kPa over the temperature range from −40 to 120 °C. As the temperature increases from −40 to 120 °C, the resonant frequency increases from 4.850 to 5.784 mHz, resulting in a frequency change of 0.934 mHz. This corresponds to a linear temperature coefficient of 5.838 kHz/°C, indicating that each 1 °C increase in temperature results in a resonant frequency shift equivalent to a pressure change of 0.24 kPa. This is a significant error, and temperature compensation is essential.

Figure 5b shows the results of constant‐pressure tests at 0.001, 100, 200, 300, 400, and 500 kPa, when the temperature is increased from −40 to 120 °C in 20 °C increments. The temperature sensitivity of the PSG resonator was measured to be 5.795, 5.789, 5.793, 5.783, 5.761, and 5.806 kHz/°C, while the TCG demonstrated temperature sensitivities of 5.608, 5.750, 5.679, 5.693, 5.683, and 5.702 kHz/°C. This close match in temperature sensitivity is highly advantageous for temperature compensation in the sensor (see Table S2, Supporting Information).

Subsequently, the pressure sensitivity of both the PSG and TCG resonators were evaluated. At constant temperatures of −40, 0, 40, 80, and 120 °C, the pressure was increased from 0.001 to 500 kPa in 40 kPa increments. The results are shown in Figure 5c. The PSG exhibited pressure sensitivities of 24.137, 24.134, 24.113, 24.141, and 24.163 kHz kPa^−1^, respectively. In contrast, the TCG showed significantly lower pressure sensitivities of 0.187, 0.185, 0.190, 0.221, and 0.195 kHz kPa^−1^. Notably, the pressure sensitivity of the TCG was two orders of magnitude lower than that of the PSG (see Table S3, Supporting Information).

To comprehensively assess the performance of the pressure sensors before and after compensation, we plotted in Figure 5d the pressure error, defined as the difference between the apparent pressure determined from Equation 2 and the calibrated pressure,^[^ ^31^ ^]^ for varying temperature and pressure. The maximum error of the compensated pressure sensor is 6.51 kPa, representing only 30.31% of the maximum error of −21.48 kPa observed before compensation. Consequently, the accuracy of our fabricated pressure sensor is quantified as 1.302%FS in the range of 0.001 to 500 kPa and the temperature range of −40 to 120 °C.

### 2.7 Comparison with Other Pressure Sensors

**Figure 6:**
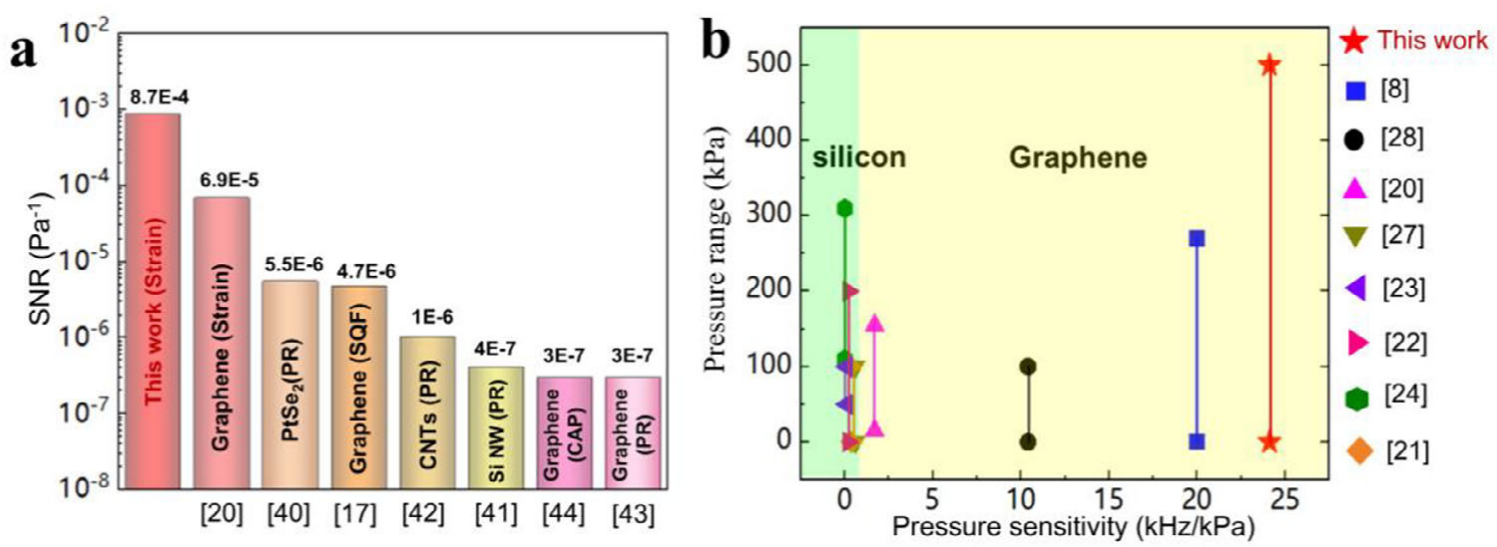
Performance comparison of resonant pressure sensors. a) SNR of piezoresistive (PR), capacitive (CAP), squeeze‐membrane (SQF) pressure sensors, and this work. b) Comparison of sensitivity and measurement range of graphene resonant pressure sensors and silicon resonant pressure sensors.

Signal‐to‐noise ratio (SNR) is an important parameter for comparing different types of pressure sensors. For the purpose of comparison, here we adopt the SNR formula per unit pressure for 2D materials from ref. [37, 38]. The SNRs of different types of representative pressure sensors (piezoresistive,^[^ ^39^, ^40^, ^41^, ^42^ ^]^ squeezed‐membrane,^[^ ^17^ ^]^ and capacitive^[^ ^43^ ^]^) are illustrated in **Figure** **6**a. Our sensor, based on the detection of resonant frequency change of the graphene membrane resulting from transmitted strain from pressure applied to the silicon diaphragm, achieves the highest SNR of 8.7 × 10^−4^ Pa^−1^, where the calculation method has been introduced in our previous work.^[^ ^20^ ^]^ Several factors together synergistically contribute to the superior SNR performance. First, the vacuum anodic bonding encapsulation creates a sealed, high‐vacuum cavity around the graphene resonators, which significantly reduces air damping and the external environmental disturbance on the resonators. This isolation enhances the stability and clarity of the resonant signal, thereby improving the SNR. Second, the sensor employs a dual‐resonator differential configuration, by measuring the differential resonant frequency between the two, temperature‐induced drift and common‐mode noise are effectively canceled out, resulting in more accurate and stable pressure detection with improved SNR. Third, depositing SiO_2_ around the edges of the graphene membrane strengthens the van der Waals interaction between the graphene and the silicon substrate. This enhanced adhesion reduces mechanical slippage and nonlinearities during vibration, minimizing signal distortion and noise.

A comparison of the sensitivity and measurement range of our pressure sensor with previous silicon resonant pressure sensors and graphene resonant pressure sensors is presented in Figure 6b. The sensitivity of our sensor is 24.1 kHz kPa^−1^, significantly exceeding the previously reported sensitivity that ranges from 0.1 to 0.353 kHz kPa^−1^ of the silicon resonant pressure sensors. At temperatures ranging from 0 to 40 °C and within the low‐pressure range of 0.001 to 5 kPa, the average sensitivity remains impressive at 25.7 kHz kPa^−1^. In addition, the maximum error is only 8.63 Pa, giving a relative accuracy is 0.173% in the pressure range of 0.001 to 5 kPa (see Figure S6, Supporting Information). The variation of measurement accuracy with the pressure can be attributed to the structural design of the pressure sensor. The PSG resonator demonstrates extremely high sensitivity to pressure changes in the low‐pressure range of 0.001 to 5 kPa, as it is located in the upper section of the silicon diaphragm, allowing it to respond quickly and accurately to slight pressure fluctuations, thereby enhancing measurement precision.

### 2.8 Application of the Pressure Sensor as a Barometric Altimeter

To demonstrate the application of a graphene resonant pressure sensor in practical situations, our sensor was mounted on a UAV to measure the flight altitude. Initially, we evaluated the minimum detectable pressure change of the sensor at a baseline pressure of 100 kPa and a temperature of 25 °C, the minimum pressure change detectable by the PSG resonator is 8.64 Pa (see Note S7, Supporting Information). The minimum detectable pressure of our sensor is better than the previously reported threshold of 90 Pa for another graphene resonator,^[^ ^8^ ^]^ the result attributed to the enhanced quality factor achieved in the vacuum encapsulated structure. Subsequently, we monitored the flight altitude of the UAV over a two‐day period under varying ambient temperatures. Given that atmospheric pressure decreases with altitude, altitude determination was facilitated through atmospheric pressure measurements (see Note S8, Supporting Information).^[^ ^44^ ^]^

**Figure 7:**
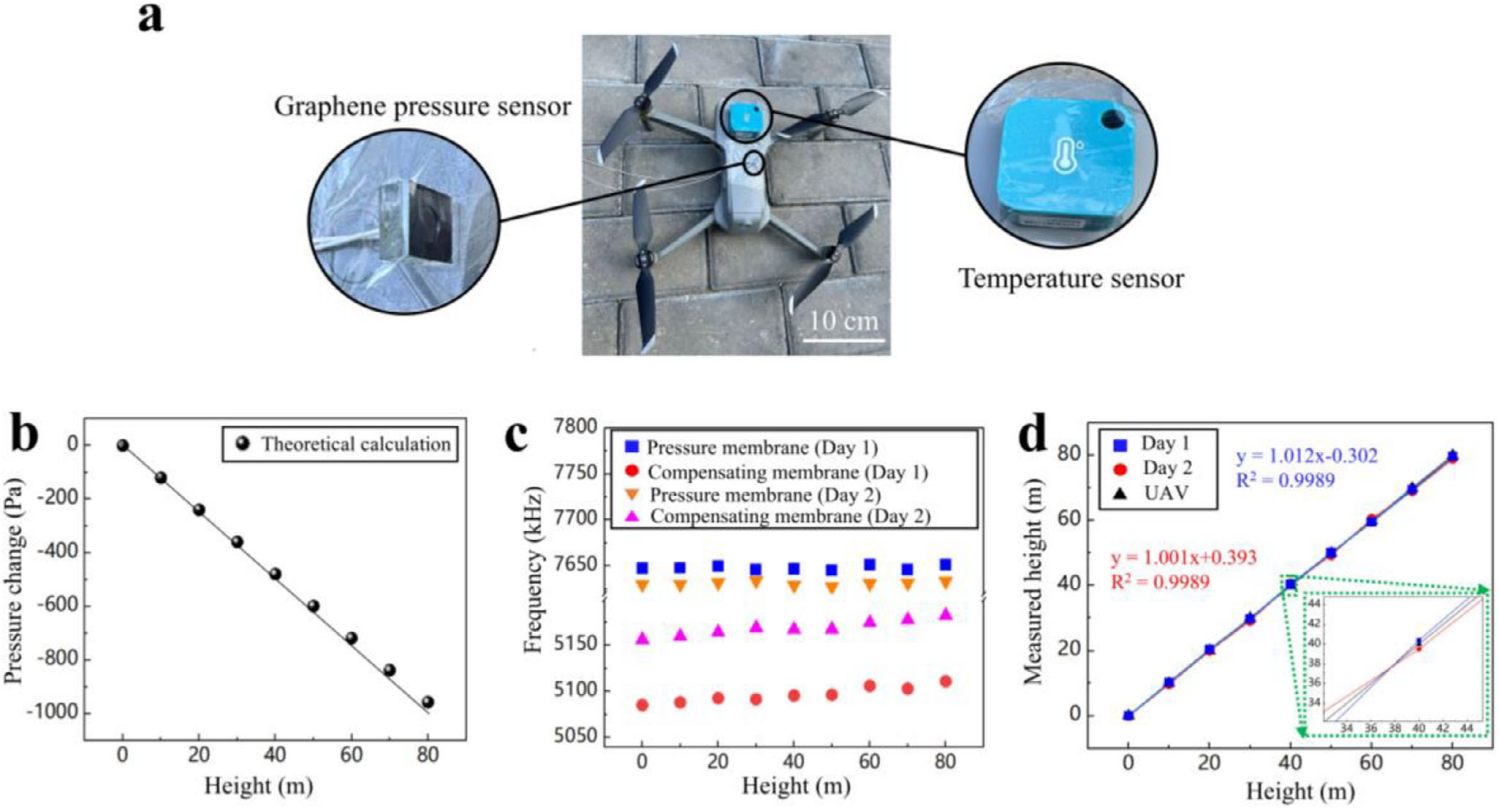
Altitude measurement with the graphene resonant pressure sensor. a) The UAV (DJI Mavic Air 2) is equipped with a graphene resonant pressure sensor and an additional commercial temperature sensor. b) The theoretical pressure variation as a function of altitude, calculated based on the polytropic atmosphere model. c) Resonant frequency of the PSG and TCG resonator vs height for two sets of measurements in two different days. Particularly, the ambient temperatures were different on the first and second day, and we recorded in steps of 10 m after the sensor output was stabilized. d) Altitude measurement from the graphene resonant pressure sensor as compared with the results from UAV commercial altimeters. The two measurements were conducted in two days.

As illustrated in **Figure** **7**a, the UAV was equipped with the graphene resonant pressure sensor in conjunction with an additional commercial temperature sensor. It should be noted that the outputs of commercial temperature sensors are provided for reference only and were excluded from the pressure result calculation. The altitude of the UAV was systematically increased from 0 to 80 m in increments of 10 m (see Figure S7, Supporting Information). As illustrated in Figure 7b, the theoretical atmospheric pressure variation with altitude was calculated using the polytropic atmosphere model. As demonstrated by the relationship defined in Equation (8.1) of Note S8 (Supporting Information), a 10‐meter altitude variation corresponds to ≈120 Pa pressure decrease. This value exceeds the sensor's minimum detectable pressure threshold of 8.64 Pa, thereby experimentally confirming the capability to resolve 10‐meter‐scale elevation changes. During the measurement process, the outputs from the PSG and TCG resonators were recorded simultaneously, with pressure values at varying heights calculated based on the sensor's pressure sensitivity. The accuracy of the measured altitudes was subsequently assessed to be ±1 m derived from the pressure‐height relationship detailed in Equation (8.1) in Note S8 (Supporting Information), as shown in Figure 7c,d. To the best of our knowledge, this study represents the first instance of a graphene resonant pressure sensor being mounted on a UAV for application as a barometric altimeter. While the developed sensor exhibits an order of magnitude lower accuracy (±1 m) compared to commercial GPS‐integrated silicon piezoresistive barometric altimeters (±0.1 m), experimental validation confirms its fundamental capability for altitude measurement applications. Furthermore, the sensor's scalability extends its applicability to various fields, including vehicle‐borne pressure monitoring, underwater pressure assessment, and soil pressure measurement, all of which are critical for meteorological predictions and geological explorations.

## 3 Discussion

Herein, we have developed a graphene resonant nanomechanical pressure sensor incorporating a differential temperature compensation structure, which demonstrates exceptional performance characterized by high sensitivity (24.1 kHz kPa^−1^), high accuracy (1.302% FS), excellent repeatability (0.75%), and low hysteresis (0.31%) in the test pressure range of 0.001 to 500 kPa and the temperature range of −40 to 120 °C. The sensor features a dual resonator structure, i.e., a PSG resonator and a TCG resonator, which effectively mitigates the influence of ambient temperature by performing differential detection. The remarkable sensitivity and stability can be largely attributed to its vacuum‐anode‐bonded encapsulated resonant structure, which seals the graphene membrane within a stable vacuum environment with its edges clamped using deposited SiO_2_ to ensure a high *Q* factor for the fundamental vibration mode. The sensitivity and pressure measuring range may be further improved by optimizing the thickness and diameter of the silicon diaphragm, allowing the sensor to meet the requirements of diverse pressure measurement environments.

We have constructed a simulation model of the graphene resonator to evaluate the pressure sensitivity of the sensor, which aligns closely with the experimental results. We have also successfully demonstrated the application of the graphene resonant pressure sensor mounted on a UAV as a barometric altimeter, achieving a height measurement accuracy of ±1 m. Our sensor provides valuable pressure and temperature information, enabling high‐precision prediction of various flight parameters when integrated with deep learning algorithms. This capability is particularly beneficial for applications in UAV navigation and control, air traffic management, and weather monitoring.^[^ ^45^, ^46^ ^]^

## 4 Conclusion 

In summary, we performed a highly sensitive graphene nanomechanical resonant pressure sensor with integrated temperature self‐compensation capability. The pressure‐sensitive graphene resonator on a thin silicon diaphragm and the temperature‐compensating graphene resonator on the thick silicon diaphragm are co‐packaged within a vacuum anode‐bonded enclosure, and the temperature dependence is removed by taking the difference between their resonance frequencies. Vacuum encapsulation and SiO_2_ edge deposition contribute to suppressing environmental perturbations, ensuring stable frequency referencing. Furthermore, a sensitivity of 24.1 kHz kPa^−1^ is achieved over the range of 0.001 to 500 kPa, which is 68 times higher than state‐of‐the‐art silicon resonant pressure sensors. The full‐scale hysteresis error is 0.31% and the repeatability is 0.75% across three bidirectional pressure sweeps. Within the test ranges of −40 to 120 °C and 0.001 to 500 kPa, the maximum pressure error is 6.51 kPa, corresponding to an accuracy of 1.302% full scale, and the temporal drift is 0.014% over 4800 s and 0.160% over 30 days. In addition, barometric altimetry with height accuracy better than ±1 m is demonstrated when the developed sensor is mounted on a UAV, underscoring the utility of differential graphene resonators for compact, high‐sensitivity pressure metrology.

## 5 Methods

### Materials

PMMA‐based graphene was purchased from Nanjing Jicang Nano Technology Co., Ltd., China. Deionized water was obtained from Beijing Keyi Mirror Trading Co., Ltd., China. Anhydrous ethanol (analytical grade) was purchased from Modern Oriental (Beijing) Technology Development Co., Ltd., China. Single‐mode fiber was purchased from Haiyu Optical Co., Ltd., China.

### FEM Simulation of Resonant Characteristics in Graphene Membrane

FEM simulation of resonant characteristics in graphene membrane was performed using the commercial software package COMSOL Multiphysics 5.6, which provides coupled multi‐physics fields from the solid mechanics and heat transfer modules, as well as allowing for the study of heat transfer. The edges of the PSG resonator were fixed, and the modelled geometry was the same as the actual measured dimensions. Under unpressurised conditions, the graphene membrane has a Young's modulus of 500 GPa, a Poisson's coefficient of 0.19, and a density of 2200 kg m^−3^ according to the previous ref. [20, 35, 36, 37].

### Vacuum Anode Bonding Packages for Pressure Sensor

The upper surface of a silicon wafer with a thickness of 500 µm was etched to form two circular holes (2 mm in diameter and 20 µm in depth). Then a secondary etching was performed within the two holes to form a micro circular pit with a diameter of 10 µm as a substrate for the graphene sensitive membrane. Next, the center of the bottom surface of the silicon wafer was etched to form a pressure‐conducting silicon diaphragm (5 mm in diameter and 100 µm in thickness). After that, the graphene/Polymethyl Methacrylate (PMMA) membrane was pressed onto the circular pits on the upper surface of the silicon diaphragm using the dry stamping method. The graphene/PMMA membrane was annealed at 350 °C under nitrogen for 3 h to remove the PMMA layer, and graphene resonators with a diameter of 10 µm were adhered to the upper surface of the silicon diaphragm by van der Waals forces. It should be noted that the multilayer rather than single‐layer graphene membrane was chosen to increase the optical reflectivity, thereby improving the visibility of the fringe pattern of the Fabry‐Perot (F–P) cavity as well as the SNR of the output signal. Moreover, SiO_2_ was deposited at the edges of the graphene resonator by the focused ion beam deposition method, which contributes to increasing the adsorption force between the graphene membrane and the silicon diaphragm and reducing the slippage of the graphene resonator during vibration (see Note S3, Supporting Information). The SiO_2_ deposition process improved the stability and hysteresis of the sensor, which has been demonstrated in the previous work.^[^ ^26^ ^]^ In this case, the silicon diaphragm was bonded anodically to a glass cap wafer adsorbed with a titanium getter to encapsulate two graphene resonators in a vacuum cavity (see Note S9, Supporting Information).

### Characterization and Mechanical Measurements

The Raman spectrum was obtained by a Raman spectrometer (LabRAM, HR Evolution, France). The thickness of graphene was measured by AFM (FSM, FM‐Nanoview 6800, China). The F–P interference spectrum was obtained by an optical spectrum analyzer (Yokogawa, AQ6370C, Japan). The deposition process of SiO_2_ and the acquisition of SEM images were performed using a helium‐neon‐gallium triple‐beam ion microscope (Carl Zeiss, Orion NanoFab, America).

In the resonance test system of the sensor, DFB lasers (KG‐DFB‐1550, Conquer Ltd., China) were used to provide the excitation and detection light. EOM (KG‐AMBOX, Beijing Conquer Optics Science & Technology Co., Ltd., Beijing, China) was used for the sinusoidal modulation of the excitation light signal. A lock‐in amplifier (HF2LI, Zurich Instruments, Switzerland) was used for the sinusoidal modulation of the output signal. EDFA (KG‐EDFA‐B, Beijing Conquer Optics Science & Technology Co., Ltd., Beijing, China) was used to amplify the excitation optical signal. WDM (CWDM, Beijing Conquer Optics Science & Technology Co., Ltd., Beijing, China) was used to filter out the excitation optical signal. A dry well calibrator (ConST670, Beijing Constant Instrument Technology Co. Ltd., Beijing, China) was used to offer a stable temperature environment for the sensor. Pressure controller (ConST810A, Beijing Constant Instrument Technology Co. Ltd., Beijing, China) was used to provide a stable pressure environment for the sensor (see Note S10 and Figure S8, Supporting Information for details).

## Conflict of Interest

The authors declare no conflict of interest.

## Author Contributions

Z.W. did all the experiments, analyzed the data, and wrote the manuscript partly. S.C.F. and P.C.Z. participated in discussing the data and editing the manuscript. Z.W.W. assisted with sensor fabrication and vacuum encapsulation. Y.L. performed finite element modelling. Z.W., P.C.Z., W.T.L., C.L., and W.J. analyzed the results and prepared the manuscript. C.L. and W.J. coordinated the project. All the authors contributed to and commented on this paper.

## Supporting information



Supporting Information

## Data Availability

The data that support the findings of this study are available from the corresponding author upon reasonable request.

## References

[1] R. Chen , T. Luo , J. Wang , R. Wang , C. Zhang , Y.u Xie , L. Qin , H. Yao , W. Zhou , Nat. Commun. 2023, 14, 6641. 37863948 10.1038/s41467-023-42361-9 PMC10589270

[2] K. Dong , Z. Wu , J. Deng , A. C. Wang , H. Zou , C. Chen , D. Hu , B. Gu , B. Sun , Z. L. Wang , Adv. Mater. 2018, 30, 1804944. 10.1002/adma.201804944 30256476

[3] Y. Zhang , S. Guo , M. Chen , B. Lu , X. Zhang , S. Liang , J. Zhou , Nat. Commun. 2023, 14, 7080. 37925505 10.1038/s41467-023-42919-7 PMC10625522

[4] C. Dagdeviren , Y. Su , P. Joe , R. Yona , Y. Liu , Y.‐S. Kim , Y. Huang , A. R. Damadoran , J. Xia , L. W. Martin , Y. Huang , J. A. Rogers , Nat. Commun. 2014, 5, 4496. 25092496 10.1038/ncomms5496

[5] T. Yamada , Y. Hayamizu , Y. Yamamoto , Y. Yomogida , A. Izadi‐Najafabadi , D. N. Futaba , K. Hata , Nat. Nanotechnol. 2011, 6, 296. 21441912 10.1038/nnano.2011.36

[6] B. Nie , R. Huang , T. Yao , Y. Zhang , Y. Miao , C. Liu , J. Liu , X. Chen , Adv. Funct. Mater. 2019, 29, 1808786.

[7] Y. Ma , N. Liu , L. Li , X. Hu , Z. Zou , J. Wang , S. Luo , Y. Gao , Nat. Commun. 2017, 8, 1207. 29089488 10.1038/s41467-017-01136-9 PMC5663936

[8] S. More , A. Naik , J. Micromech. Microeng. 2024, 34, 075006.

[9] R. A. Barton , B. Ilic , A. M. van der Zande , W. S. Whitney , P. L. McEuen , J. M. Parpia , H. G. Craighead , Nano Lett. 2011, 11, 1232. 21294522 10.1021/nl1042227

[10] B. Xu , P. Zhang , J. Zhu , Z. Liu , A. Eichler , X.u‐Q. Zheng , J. Lee , A. Dash , S. More , S. Wu , Y. Wang , H. Jia , A. Naik , A. Bachtold , R. Yang , P. X.‐L. Feng , Z. Wang , ACS Nano 2022, 16, 15545. 36054880 10.1021/acsnano.2c01673 PMC9620412

[11] D. Moreno‐Garcia , X. Fan , A. D. Smith , M. C. Lemme , V. Messina , C. Martin‐Olmos , F. Niklaus , L. G. Villanueva , Small 2022, 18, 2201816. 10.1002/smll.202201816 35638191

[12] A. Blaikie , D. Miller , B. J. Alemán , Nat. Commun. 2019, 10, 4726. 31624243 10.1038/s41467-019-12562-2 PMC6797740

[13] T. Cui , S. Mukherjee , P. M. Sudeep , G. Colas , F. Najafi , J. Tam , P. M. Ajayan , C. V. Singh , Y. Sun , T. Filleter , Nat. Mater. 2020, 19, 405. 31959950 10.1038/s41563-019-0586-y

[14] P. Zhang , L. Ma , F. Fan , Z. Zeng , C. Peng , P. E. Loya , Z. Liu , Y. Gong , J. Zhang , X. Zhang , P. M. Ajayan , T. Zhu , J. Lou , Nat. Commun. 2014, 5, 3782. 24777167 10.1038/ncomms4782

[15] R. R. Nair , P. Blake , A. N. Grigorenko , K. S. Novoselov , T. J. Booth , T. Stauber , N. M. R. Peres , A. K. Geim , Science 2008, 320, 1308. 18388259 10.1126/science.1156965

[16] J. Ma , W. Jin , H. Xuan , C. Wang , H. L. Ho , Opt. Lett. 2014, 39, 4769. 25121870 10.1364/OL.39.004769

[17] R. J. Dolleman , D. Davidovikj , S. J. Cartamil‐Bueno , H. S. J. van der Zant , P. G. Steeneken , Nano Lett. 2016, 16, 568. 26695136 10.1021/acs.nanolett.5b04251

[18] R. J. Dolleman , D. Chakraborty , D. R. Ladiges , H. S. J. van der Zant , J. E. Sader , P. G. Steeneken , Nano Lett. 2021, 21, 7617. 34461013 10.1021/acs.nanolett.1c02237 PMC8461654

[19] M. Lee , D. Davidovikj , B. Sajadi , M. Siskins , F. Alijani , H. S. J. van der Zant , P. G. Steeneken , Nano Lett. 2019, 19, 5313. 31340117 10.1021/acs.nanolett.9b01770 PMC6696884

[20] Y. Liu , C. Li , X. Shi , Z. Wu , S. Fan , Z. Wan , S. Han , ACS Appl. Mater. Interfaces 2023, 15, 30479. 37307273 10.1021/acsami.3c04520

[21] J. B. Wang , D. Y. Chen , L. Liu , Z. W. Wu , in A Micromachined Resonant Pressure Sensor with DETFs Resonator and Differential Structure , IEEE, Christchurch, New Zealand 2009.

[22] X. Han , Q.i Mao , L. Zhao , X. Li , L.i Wang , P. Yang , D. Lu , Y. Wang , X. Yan , S. Wang , N. Zhu , Z. Jiang , Microsyst. Nanoeng. 2020, 6, 95. 34567705 10.1038/s41378-020-00207-0 PMC8433135

[23] Z. Y. Luo , D. Y. Chen , J. B. Wang , Y. N. Li , J. Chen , Sensors 2014, 14, 24244. 25521385 10.3390/s141224244 PMC4299109

[24] Y. D. Li , Y. L. Lu , B. Xie , J. Chen , J. B. Wang , D. Y. Chen , IEEE Trans. Electron. Devices 2020, 67, 640.

[25] Z. Wan , C. Li , Y. Liu , Y. J. Liu , X. Xiao , S. Han , Appl. Surf. Sci. 2023, 639, 158237.

[26] Z. Wan , C. Li , Z. Wu , Y. Liu , R. Liu , W. Zhou , Q. Wang , ACS Appl. Mater. Interfaces 2024, 16, 38792. 38980283 10.1021/acsami.4c08045

[27] Z. Wan , C. Li , S. Lu , Y. Liu , Y. Liu , S. Fan , S. Han , IEEE Sens. J. 2023, 23, 22332.

[28] B.o Xu , J. Zhu , F. Xiao , N.a Liu , Y. Liang , C. Jiao , J. Li , Q. Deng , S. Wu , T. Wen , S. Pei , H. Wan , X.u Xiao , J. Xia , Z. Wang , ACS Nano 2022, 16, 20229. 36508311 10.1021/acsnano.2c05742

[29] R. J. Dolleman , D. Lloyd , M. Lee , J. S. Bunch , H. S. J. van der Zant , P. G. Steeneken , Phys. Rev. Mater. 2018, 2, 114008.

[30] Y. Chen , S. Liu , G. Hong , M. Zou , B. Liu , J. Luo , Y. Wang , ACS Appl. Mater. Interfaces 2022, 14, 39211. 35994410 10.1021/acsami.2c09865 PMC9438774

[31] Z. Y. Tang , S. C. Fan , W. W. Xing , Z. S. Guo , Z. Y. Zhang , Microsyst. Technol. 2011, 17, 1481.

[32] D. Davidovikj , M. Poot , S. J. Cartamil‐Bueno , H. S. J. van der Zant , P. G. Steeneken , Nano Lett. 2018, 18, 2852. 29653051 10.1021/acs.nanolett.7b05358 PMC6023267

[33] H. Y. Choi , K. S. Park , S. J. Park , U. C. Paek , B. H. Lee , E. S. Choi , Opt. Lett. 2008, 33, 2455. 18978885 10.1364/ol.33.002455

[34] J. Wang , X. Wei , J. Shi , N. Bai , X. Wan , B. Li , Y. Chen , Z. Jiang , C. F. Guo , Nat. Commun. 2024, 15, 7094. 39153996 10.1038/s41467-024-51415-5 PMC11330526

[35] P. F. Ferrari , S. Kim , A. M. van der Zande , Nano Lett. 2021, 21, 8058. 34559536 10.1021/acs.nanolett.1c02369

[36] C. Chen , S. Rosenblatt , K. I. Bolotin , W. Kalb , P. Kim , I. Kymissis , H. L. Stormer , T. F. Heinz , J. Hone , Nat. Nanotechnol. 2009, 4, 861. 19893525 10.1038/nnano.2009.267

[37] J. S. Bunch , A. M. van der Zande , S. S. Verbridge , I. W. Frank , D. M. Tanenbaum , J. M. Parpia , H. G. Craighead , P. L. McEuen , Science 2007, 315, 490. 17255506 10.1126/science.1136836

[38] B. Blankertz , R. Tomioka , S. Lemm , M. Kawanabe , K. R. Müller , IEEE Signal Process. Mag. 2008, 25, 41.

[39] S. Wagner , C. Yim , N. McEvoy , S. Kataria , V. Yokaribas , A. Kuc , S. Pindl , C.‐P. Fritzen , T. Heine , G. S. Duesberg , M. C. Lemme , Nano Lett. 2018, 18, 3738. 29768010 10.1021/acs.nanolett.8b00928 PMC6014683

[40] C. Stampfer , T. Helbling , D. Obergfell , B. Schöberle , M. K. Tripp , A. Jungen , S. Roth , V. M. Bright , C. Hierold , Nano Lett. 2006, 6, 233. 16464041 10.1021/nl052171d

[41] J. H. Zhang , Y. Zhao , Y. X. Ge , M. Li , L. J. Yang , X. L. Mao , Micromachines 2016, 7, 187. 30404360 10.3390/mi7100187 PMC6189815

[42] Q. G. Wang , W. Hong , L. Dong , Nanoscale 2016, 8, 7663. 26988111 10.1039/c5nr09274d

[43] D. Davidovikj , P. H. Scheepers , H. S. J. van der Zant , P. G. Steeneken , ACS Appl. Mater. Interfaces 2017, 9, 43205. 29164848 10.1021/acsami.7b17487

[44] M. Chen , W. Luo , Z. Xu , X. Zhang , B.o Xie , G. Wang , M. Han , Nat. Commun. 2019, 10, 4024. 31492843 10.1038/s41467-019-12030-x PMC6731318

[45] G. E. Karniadakis , I. G. Kevrekidis , L. Lu , P. Perdikaris , S. F. Wang , L. Yang , Nat. Rev. Phys. 2021, 3, 422.

[46] B. M. Lake , R. Salakhutdinov , J. B. Tenenbaum , Science 2015, 350, 1332. 26659050 10.1126/science.aab3050

