# Supplementary material for: Ultra‐Sensitive Dual‐Resonator Graphene Pressure Sensor with Temperature Self‐Compensation
Source: Adv Sci (Weinh). 2025 Nov 5;13(5):e17536. doi: 10.1002/advs.202517536 (PMC12850379; doi:10.1002/advs.202517536)
Supplement: Supplementary file 1 — Supporting Information [file ADVS-13-e17536-s001.docx]

**Supporting information**

Ultra-sensitive dual-resonator graphene pressure sensor with temperature self-compensation

*Wan et.al.*

# Supplementary Note 1: Photothermal excitation/detection principle for graphene resonant sensor

The schematic diagram of the resonator for photothermal excitation and detection is depicted in Supplementary Fig. 1a, illustrating a low-fineness Fabry-Perot (F-P) interference cavity formed between the end face of a single-mode fiber (SMF) and a graphene membrane. When a sinusoidally modulated incident laser irradiates the membrane's surface, a portion of the light is absorbed, converting optical energy into thermal energy. This absorption generates a temperature field and a corresponding strain field that vary periodically at the same frequency of laser modulation. Consequently, the membrane undergoes expansion and contraction at this frequency, resulting in periodic vibration, as shown in Supplementary Fig. 1b. Specifically, when the incident light is emitted from the SMF, Fresnel reflection occurs at the end face of optical fiber due to the refractive index difference between the SMF and the surrounding environment, resulting the formation of a reflected beam. The remaining light subsequently propagates freely to the graphene membrane, where a second Fresnel reflection occurs at the reflected surface, resulting in another reflected beam.

When the pressures on the two sides of the membrane is different, the graphene membrane produces a flexural deformation. The tension inside the graphene membrane is composed of two parts: the initial tension *S*_0_ of the membrane and the tension *S*_p_ formed by the membrane under the action of the pressure as

$S_{\text{p}}=\sigma_{\text{p}}t_{\text{g}}=\frac{E_{\text{g}}\varepsilon_{\text{p}}}{1-\mu_{\text{g}}}t_{\text{g}}=\frac{2E_{\text{g}}t_{\text{g}}w_{\text{g}}^{2}}{3r_{\text{g}}^{2}\left(1-\mu_{\text{g}}\right)}$ (1.1)

$S_{0}=\sigma_{0}t_{\text{g}}$ (1.2)

where *E*_g_, *μ*_g_, *t*_g_, *r*_g_ and *w*_g_ are the Young's modulus, the Poisson's ratio, the thickness, the radius and the deflection deformation of graphene membrane, respectively; *σ*_p_ and *ε*_p_ are the additional stress and additional strain of graphene membrane under pressure, respectively, and *σ*_0_ is the prestress of graphene membrane.

The total tension *S*_g_ of the graphene membrane is related to the pressure *p*, which can be written as

$S_{\text{g}}=S_{\text{p}}+S_{0}=\frac{2E_{\text{g}}t_{\text{g}}h_{\text{g}}^{2}}{3r_{\text{g}}^{2}\left(1-\mu_{\text{g}}\right)}+S_{0}=\frac{pr_{\text{g}}^{2}}{4h_{\text{g}}}$ (1.3)

where *h*_g_ is the maximum deflection deformation of graphene membrane. Then based on the equations (1.2) ~ (1.4), the pressure *p* can be expressed as

$p=\frac{8E_{\text{g}}t_{\text{g}}h_{\text{g}}^{3}}{3r_{\text{g}}^{4}\left(1-\mu_{\text{g}}\right)}+\frac{4S_{0}h_{\text{g}}}{r_{\text{g}}^{2}}=\frac{8E_{\text{g}}t_{\text{g}}h_{\text{g}}^{3}}{3r_{\text{g}}^{4}\left(1-\mu_{\text{g}}\right)}+\frac{4\sigma_{0}t_{\text{g}}h_{\text{g}}}{r_{\text{g}}^{2}}$ (1.4)

The fundamental frequency of the graphene resonator is given by^2^

$f=\frac{2.404}{2\text{π}r_{\text{g}}}\sqrt{\frac{S_{\text{g}}}{\rho_{\mathrm{g}}t_{\text{g}}}}$ (1.5)

where *ρ*_g_ is the mass per unit area of graphene membrane.

# Supplementary Note 2: Temperature compensation principle for the pressure sensor

The present sensor adopts a differential temperature compensated structure with dual graphene resonators, where the PSG resonator is located in the pressure-sensitive region and is sensitive to both the applied pressure and ambient temperature, while the TCG resonator is only sensitive to temperature. The first-mode resonant frequency *f*_1_ of the PSG resonator under pressure *p* can be expressed as^3^

$f_{1}(p)=f_{0}+kp$ (2.1)

where *k* and *f*_0_ are the pressure sensitivity and fundamental frequency of the PSG resonator, respectively. However, in practice, in addition to the pressure to be measured, other factors can also cause stress changes in the graphene resonator, mainly including thermal stress caused by ambient temperature and laser irradiation, and residual stress introduced during processing. Therefore, combining the above factors, the resonant frequency expression of PSG resonator is obtained as follows

$f_{\text{r1}}=f_{01}\text{+}k_{\text{b}1}k_{\text{p1}}p+k_{\text{b}1}k_{\text{ΔT1}}\Delta T+k_{\text{b1}}k_{\text{w}1}P_{\text{laser}}\text{+}k_{\text{b1}}\sigma_{\text{res1}}$ (2.2)

where *f*_r1_ and *f*_01_ are the first-mode resonant frequency and initial frequency of the PSG resonator; *k*_b1_ and *k*_p1_ are the stress sensitivity coefficient and pressure sensitivity coefficient of the PSG resonator;$\sigma_{\text{res1}}$ is the residual stress introduced in the process of membrane preparation and transfer; $k_{\text{ΔT1}}$ and $k_{\text{w}1}$ are the stress sensitivity coefficients of the PSG resonator due to ambient temperature change$\Delta T$and laser thermal irradiation *P*_laser_, respectively.

Similarly, the resonant frequency of the TCG resonator can be expressed as

$f_{\text{r2}}=f_{02}\text{+}k_{\text{b2}}k_{\text{p2}}p+k_{\text{b2}}k_{\text{ΔT2}}\Delta T+k_{\text{b2}}k_{\text{w2}}P_{\text{laser}}\text{+}k_{\text{b2}}\sigma_{\text{res2}}$ (2.3)

where *f*_r2_ and *f*_02_ are the first-mode resonance frequency and initial frequency of the TCG resonator; *k*_b2_ and *k*_p2_ are the stress sensitivity coefficient and pressure sensitivity coefficient of the TCG resonator;$\sigma_{\text{res2}}$is the residual stresses introduced in the process of membrane preparation and transfer; $k_{\text{ΔT2}}$ and $k_{\text{w2}}$ are the stress sensitivity coefficients of the TCG resonator due to ambient temperature change$\Delta T$and laser thermal irradiation *P*_laser_, respectively.

During the actual operation of the sensor, the excitation optical power is limited and kept constant in order to ensure that the graphene resonator works in a linear vibration state. Also, the residual stresses in the PSG resonator and the TCG resonator after the membrane preparation and transfer are regarded as the fixed values, thereby the following parameters are defined:

$\left\{\begin{matrix} \overline{f_{\text{0n}}}=f_{0n}+k_{bn}k_{wn}P_{stat}\text{+}k_{bn}\sigma_{resn} \\ k_{n}=k_{bn}k_{pn} \\ s_{n}=k_{bn}k_{\Delta Tn} \end{matrix}\right.$ (2.4)

where *n*=1, 2 (corresponding to PSG and TCG, respectively); $\overline{f_{01}}$and $\overline{f_{02}}$are the equivalent intrinsic frequencies of the resonator containing the effect of the excitation optical power and residual stress, respectively, which can be considered as the initial frequencies of the two graphene resonators during calibration; *k*_1_, *k*_2_ and *s*_1_, *s*_2_ are the pressure sensitivities and the temperature sensitivities of the PSG resonator and the TCG resonator, respectively.

Therefore, the resonant frequencies of the PSG resonator and the TCG resonator can be expressed as the following equations

$\left\{\begin{matrix} f_{r1}\text{=}\overline{f_{01}}+k_{1}p\text{+}s_{1}\Delta T \\ f_{r2}\text{=}\overline{f_{02}}+k_{2}p\text{+}s_{2}\Delta T \end{matrix}\right.$ (2.5)

From the above equations, the pressure *p* can be confirmed as:

$p=\frac{s_{2}(f_{r1}-\overline{f_{01}})-s_{1}(f_{r2}-\overline{f_{02}})}{k_{1}s_{2}-k_{2}s_{1}}$ (2.6)

# Supplementary Note 3: Vacuum anodic bonding package for the pressure sensor

As shown in Supplementary Fig. 2, the process began with etching the upper surface of a silicon wafer, which was 500 µm thick, to create two circular regions, each with a diameter of 2 mm and a depth of 20 µm. Subsequently, a secondary etching was conducted within these areas to form micro circular pits with a diameter of 10 μm, which served as the substrate for the PSG resonator. The center of the bottom surface of the silicon wafer was then etched to form a pressure-conducting silicon diaphragm with a diameter of 5 mm and a thickness of 100 µm. The graphene/Polymethyl Methacrylate (PMMA) membrane was pressed onto the circular pits on the upper surface of the silicon diaphragm using the dry stamping method. The membrane was annealed at 350 °C under nitrogen for 3 hours to remove the PMMA layer, allowing the graphene resonators, with a diameter of 10 μm, to adhere to the upper surface of the silicon diaphragm through van der Waals forces. Additionally, SiO_2_ was deposited at the edges of the graphene resonators using the focused ion beam deposition method, which enhanced the adhesion between the graphene resonator and the silicon diaphragm, mitigating slippage during vibration. The SiO_2_ deposition significantly improved the stability and reduced hysteresis of the sensor, as demonstrated in our previous work. In this configuration, the silicon diaphragm was anodically bonded to a glass cap wafer coated with titanium getter, creating a vacuum cavity to encapsulate the two graphene resonators. Specifically, the PSG resonator was positioned at the center of the silicon diaphragm, while the TCG resonator was located at its edge.

Supplementary Fig. 3 illustrates the fabrication process of the graphene resonator. The graphene membrane was obtained by transferring a ~10-layered graphene sample from commercial sources, as depicted in Supplementary Fig. 3a. Notably, the multilayer graphene (MLG) membrane is affixed to a PMMA substrate. Subsequently, the PMMA/MLG composite material was adhered to a 10 μm diameter hole on the silicon substrate using the wet transfer method, as shown in Supplementary Figs. 3b and 3c. Finally, the PMMA/MLG/Si structure was annealed in an inert nitrogen environment at 350 °C to remove the PMMA, thereby fabricating the graphene resonator, as illustrated in Supplementary Fig. 3d.

# Supplementary Note 4: Assembling the optical fiber and silicon diaphragm with glass block

This section outlines the experimental setup for the integration of optical fiber and the graphene resonator. As illustrated in Supplementary Fig. 4, a broadband laser and a red laser with a center wavelength of 650 nm were coupled through a 50/50 coupler. The combined light was transmitted through a circulator and directly into the sealed resonator, where the fiber tip was positioned inside a glass capillary mounted on a motorized *x-y-z* stage. This configuration facilitated precise positioning of the laser spot on the graphene resonator with a spatial resolution of 1 μm. The reflected light was subsequently coupled back into the fiber and transmitted through the circulator to an optical spectrum analyzer (OSA), which featured a high wavelength resolution of 0.02 nm. After adjusting the laser spot to the center of the graphene membrane, the capillary and glass cap wafer were bonded together.

# Supplementary Note 5: Optical vibration detection scheme

This section describes the vibration detection scheme based on multi-beam interference, which is related to the spatial spreading of light. A schematic diagram of multi-beam interference of the sensor probe used is shown in Supplementary Fig. 5a, wherein the optical power of transmitted light through each surface are respectively represented by ***E***_0_′, ***E***_0_″′and ***E***_0_‴, while the reflected light power by each surface are represented by ***E***_1_, ***E***_2_, ***E***_3_ and ***E***_4_, respectively. It should be noted that due to the structural design of the sensor, the light transmitted through the optical fiber is considered to be perpendicular to the sealed resonator.

The derivation of multi-beam interference for this sensor configuration can be found in our previous work^4^. Based on this theoretical framework, the interference spectrum can be calculated by defining the reflectivity and distances between reflected surfaces, with structural parameters including a 135 μm gap between reflected Surfaces 1 and 2, 200 μm between Surfaces 2 and 3 and 10 μm between Surfaces 3 and 4, coupled with reflectivities of 4%, 4%, 4%, and 1.5% for Surfaces 1~4 respectively. Mathematical modeling of the interference spectrum enables to optimize the sensor design and improve the detection accuracy. In Supplementary Fig. 5b, the red square marks the 1550 nm laser wavelength (commonly used in optical communications), where the steep slope indicates high sensitivity of light intensity to cavity length variations at this wavelength. Selecting this operating point enhances vibration detection sensitivity. The near-identical reflectivities of Surfaces 1-3 confirm that optical interference between Surface 3 (glass substrate) and Surface 4 (pressure-sensitive graphene, PSG resonator) dominates the spectral response. In Supplementary Fig. 5b, the red square marks the 1550 nm laser wavelength (commonly used in optical communications), where the steep slope indicates high sensitivity of light intensity to cavity length variations at this wavelength. Selecting this operating point enhances vibration detection sensitivity.

Supplementary Fig. 5c displays multi-beam interference spectra for both the PSG resonator and temperature-compensated graphene (TCG) resonator, characterizing the F-P cavity lengths. Under illumination from a 1520~1600 nm broadband light source, light reflected from multiple surfaces (fiber end-face, glass cover plate, and graphene resonators) generates interference signals. With a glass cover plate thickness of 150 µm, demodulation yields fiber-to-glass distances of 137 µm (PSG) and 132 µm (TCG), corresponding to total F-P cavity lengths of 287 µm and 282 µm, respectively. While simulations confirm dominance of interference between the graphene resonators and glass substrate, experimental spectra exhibit non-ideal multi-beam interference patterns due to unavoidable contributions from other surfaces. However, this non-ideality does not affect resonant frequency demodulation in the designed sensor structure.

# Supplementary Note 6: Repeatability of pressure sensing in graphene resonator

To demonstrate the repeatability of the sensor, the experimental results from three forward and backward cycles are evaluated, and the repeatability is quantified using the range method

$W_{\text{i}}=\max(y_{\text{ij}})-\min(y_{\text{ij}}),j=1,2,\text{3，}\cdots$ (6.1)

where *W*_i_ is the range, denoting the difference between the maximum and minimum values of the calibrated values at the *i^th^* measurement point.

$s_{\text{i}}=\frac{W_{\text{i}}}{d_{\text{m}}}$ (6.2)

where *s*_i_ is the standard deviation of the *i^th^* measurement point; *d*_m_ is the range coefficient, which depends on the sample size. Since three cycles of measurements are conducted, *d*_m_ = 1.91^4^.

$s=\sqrt{\frac{1}{n}\sum_{i=1}^{n} s_{\text{i}}^{2}}$ (6.3)

where *s* is the standard deviation of the whole measurement process. Therefore, the repeatability of the sensor can be given by

$\xi_{R}=\frac{3s}{y_{\text{FS}}}\times100\%$ (6.4)

where *y*_FS_ is the full-scale output of the sensor.

# Supplementary Note 7: The calculation of the minimum detectable pressure

In resonant pressure sensors, the responsivity (R) is defined as the change in resonance frequency per unit change in pressure (R = Δf/ΔP). It is important to note that in literature related to MEMS pressure sensors, this term is often referred to as sensitivity [3-5]. The resolution of a pressure sensor represents the minimum detectable change in pressure. For resonant pressure sensors, the resolution is calculated as the product of the minimum detectable frequency change and the inverse of the responsivity.

$\Delta p_{\min}=\Delta f\times R^{-1}$ (7.1)

In this work, the minimum detectable frequency change is 208 Hz, and the responsivity is 24.1 Hz/Pa. Therefore, the minimum detectable pressure is 8.64 Pa.

# Supplementary Note 8: The calculation of altitude pressure

In the barometric altimeter experiments carried by the UAV, the pressure measurement of the developed sensor versus altitude can be calculated from a multidirectional atmospheric model. To perform the calculation, the relationship between pressure and altitude is established from a polytropic atmosphere model (See: website of the National Oceanic and Atmospheric Administration (NOAA) (https://www.weather.gov/media/epz/wxcalc/pressureAltitude.pdf)), as shown in the following equation^5^

$p_{\text{ALT}}=101325\times(1-\frac{h}{0.3048\times145366.45})^{\frac{1}{0.190284}}$ (8.1)

where *p*_ALT_ is the barometric pressure at the altitude *h*.

# Supplementary Note 9: SiO_2_ deposition and vacuum anode bonding

The SEM images were taken by a helium-neon-gallium triple-beam ion microscope. Notably, a helium ion beam of 0.5 nm @30 kV for SiO_2_ deposition with a beam current of 1 pA was used. Moreover, the edge deposition did not damage the graphene membrane as can be seen from the focused position image of SiO_2_ deposition. Graphene resonant pressure sensors were packaged by vacuum anode bonding. In this work, the anode bonding was carried out within an ambient pressure of 5×10^-3^ Pa at a temperature of 350°C, an electric field voltage of 1000 V, and a bonding time of 30 min.

# Supplementary Note 10: Experimental environment and protection of the sensor

To ensure reliable sensor probe functionality and optical fiber protection under experimental conditions, a customized experimental configuration (Supplementary Fig. 8a) was developed, including a vacuum chamber integrated with pressure and temperature controllers. The customized chamber design isolates the sensor probe within controlled environmental parameters while shielding the optical fiber from extreme thermal and pressure exposure. Critical interface protection at the fiber-probe junction (Supplementary Fig. 8b) employs high-temperature resistant adhesive (AB-Kleber, Germany) bonding between the fiber clamp and pressure-sensitive silicon chip, maintaining structural integrity from -40 ℃ to 200°C, supplemented by thermal insulation through aluminum silicate fiber bulk wool packing and outer encapsulation using polytetrafluoroethylene (PTFE) high-temperature tape (300 °C rated). This multi-layered structure enables precise environmental regulation of the probe while preventing thermal degradation at critical connections. For vacuum chamber integration, optical fibers were strategically cut off, threaded through dedicated feedthroughs, and subsequently reconnected externally via fusion-spliced FC/APC connectors, ensuring uninterrupted signal transmission while maintaining chamber integrity throughout thermal and pressure testing process.


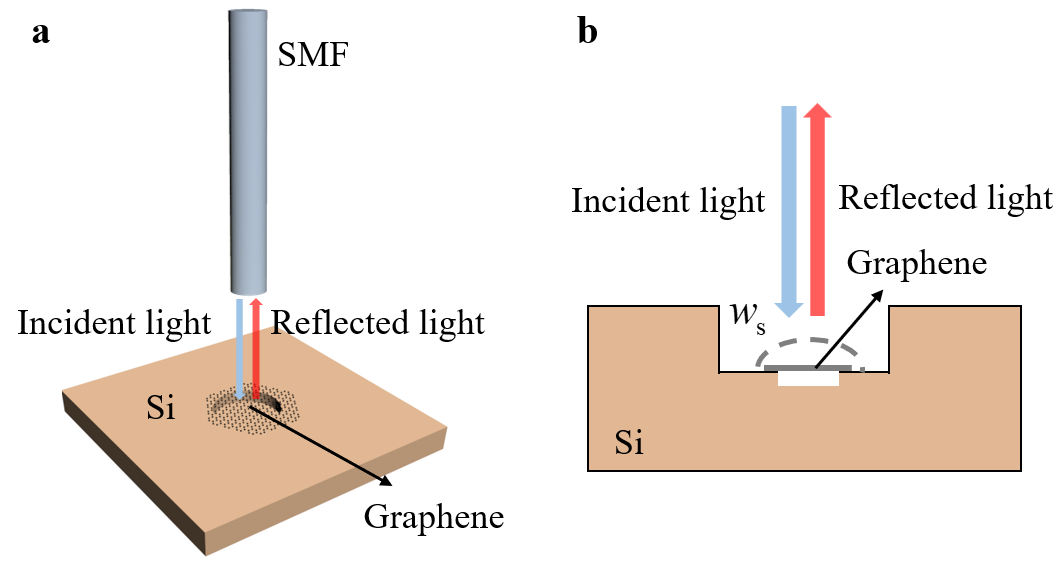


**Supplementary Fig. 1 a** Schematic diagram of excitation/detection of graphene resonator via an optical fiber. **b** Schematic diagram of a graphene membrane under photothermal excitation.


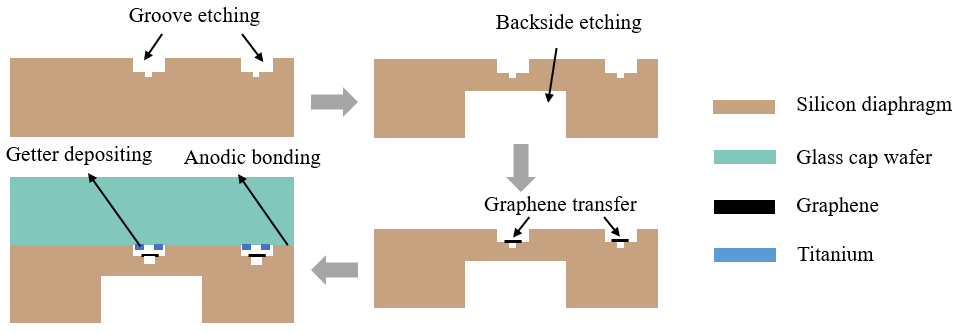


**Supplementary Fig. 2** Fabrication process of the resonant pressure sensor. Firstly, the upper surface of the silicon wafer was etched to form two circular pits for supporting the suspended graphene resonator. Then, the lower surface of the silicon wafer was etched to form a pressure sensitive diaphragm. Subsequently, the graphene membrane was transferred to cover the circular pits on the upper surface of the silicon wafer by dry transfer. Finally, a titanium getter was deposited and then the glass cap wafer and pressure sensitive silicon diaphragm were vacuum anodically bonded.


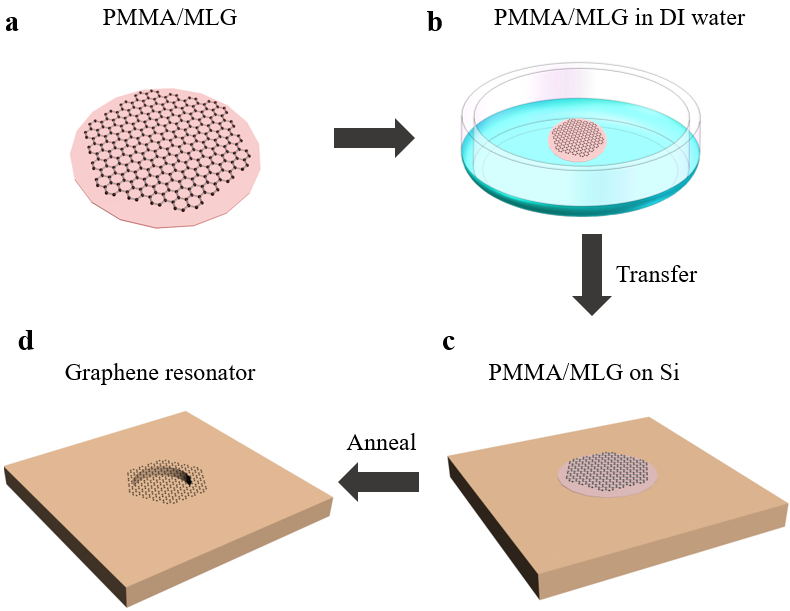


**Supplementary Fig. 3** Schematic diagram of graphene transfer process. **a** MLG on the PMMA substrate. **b** PMMA/MLG immersed in deionized (DI) water. **c** PMMA/MLG was transferred to Si substrate. **d** Graphene resonator after removal of PMMA substrate by annealing


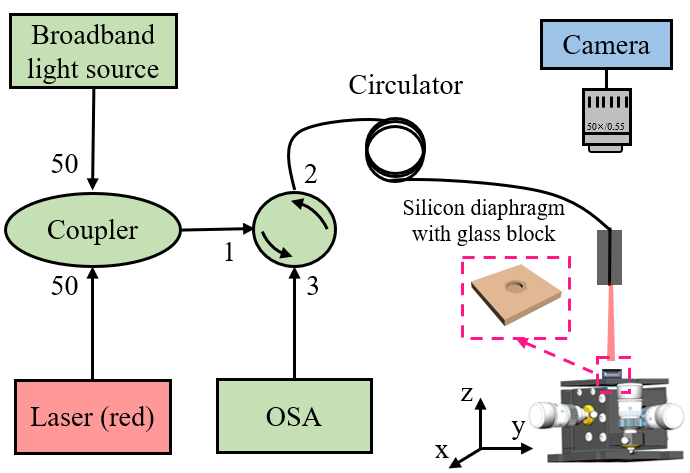


**Supplementary Fig. 4** The experimental setup of assembling the optical fiber and silicon diaphragm with glass cap wafer.


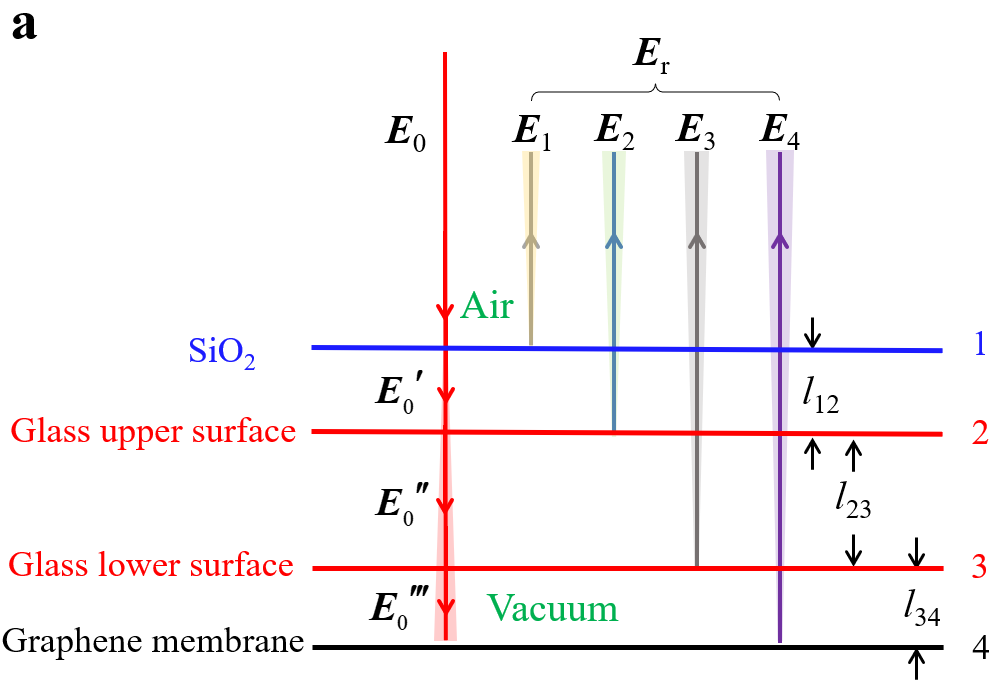

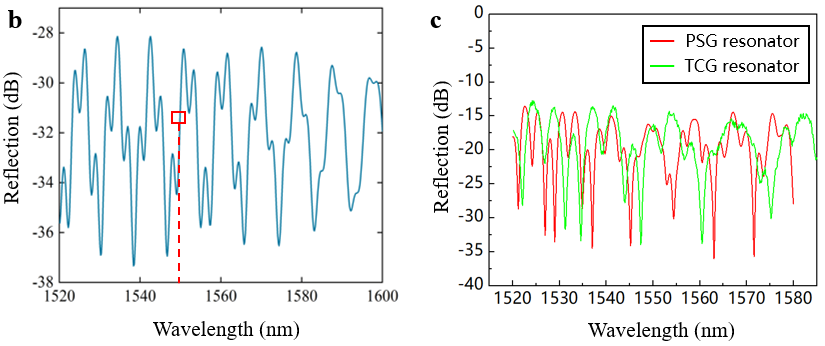


**Supplementary Fig. 5** **a** Schematic diagram of multi-beam interference of the pressure sensor. **b** Simulation result of multi-beam interference spectrum of sensor structure. **c** Experimental results of multi-beam interference spectrum of sensor structures

**
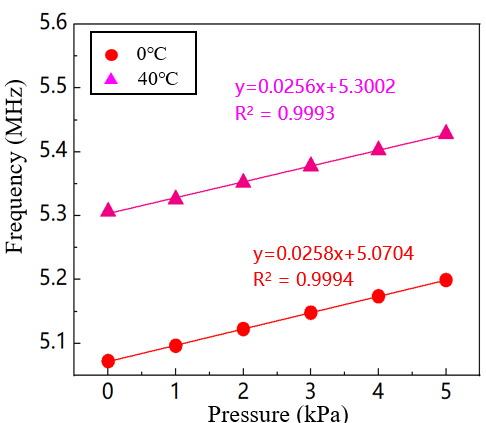
**

**Supplementary Fig. 6** Resonant frequency versus pressure for the PSG resonator at different temperatures (0 °C, 40 °C)


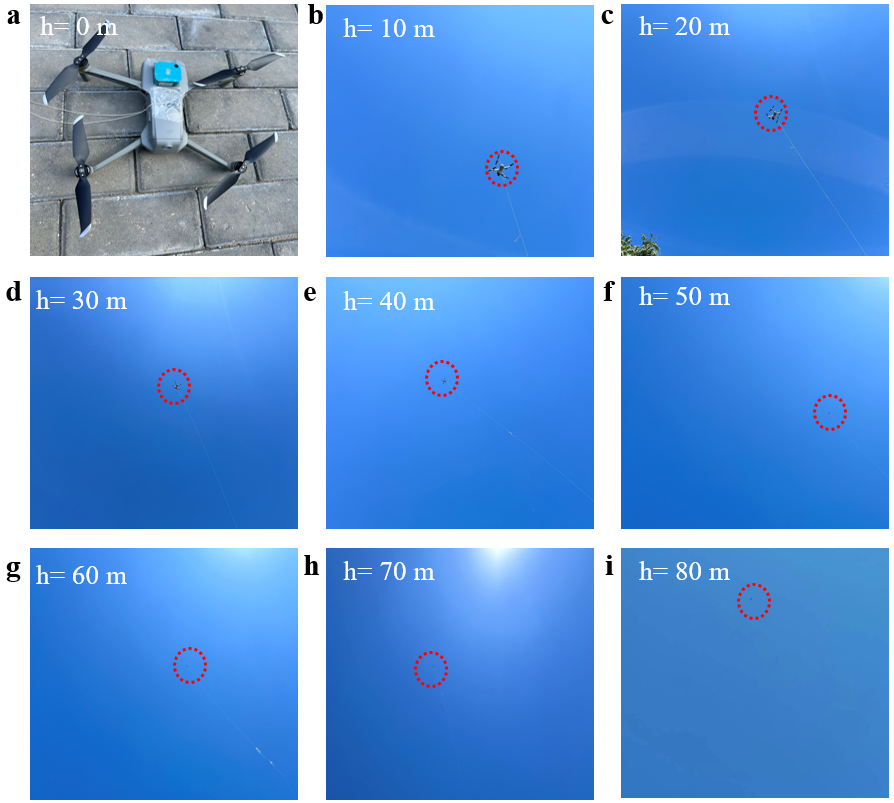


**Supplementary Fig. 7** Photos of unmanned aerial vehicle carrying the sensor during flight (altitude ranging from 0 m to 80 m). The two lines to the aircraft are optical fibers, each assigned to one resonator (PSG and TCG, respectively).


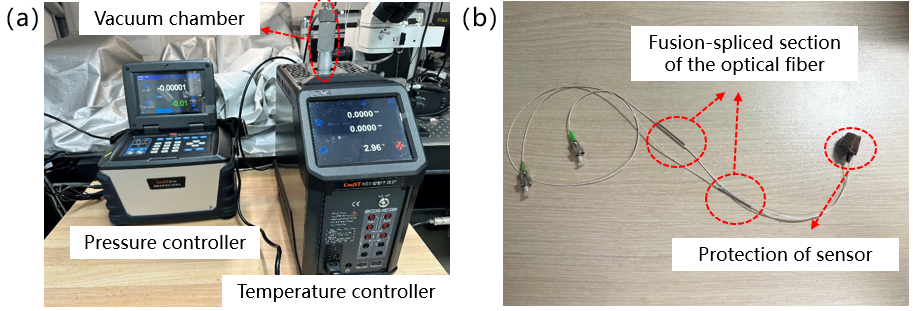


**Supplementary Fig. 8** **a** Pressure-temperature experimental system for the pressure sensor. **b** Protective measures of the pressure sensor.

**Supplementary Table 1.** The finite element simulation parameters for the photothermal properties of graphene (The parameters are derived from Refs. 6-9).

| Parameter | Value |
| --- | --- |
| Laser power (excitation and detection) | 4.8 mW |
| Graphene thickness | 6.6 nm |
| Young's modulus of graphene**^3^** | 300 GPa |
| Light absorption coefficient of graphene**^3^** | 2.3% |
| Graphene density**^3^** | 2208 kg/m^3^ |
| Graphene Poisson's ratio**^3^** | 0.19 |
| Graphene thermal conductivity**^3^** | 5300 W/(m·K) |
| Graphene thermal expansion coefficient**^3^** | -7×10^-6^ K^-1^ |
| Diameter of graphene membrane | 10 μm |
| Tension within graphene membrane | 0.07 N/m |

**Supplementary Table 2.** The temperature sensitivities of the PSG resonator and the TCG resonator at different pressures. The temperature range is from -40 ℃ to 120 ℃.

| Pressure (kPa) | PSG resonator *s*_1_ (kHz/℃) | TCG resonator *s*_2_ (kHz/℃) |
| --- | --- | --- |
| 0.001 | 5.795 | 5.608 |
| 100 | 5.789 | 5.750 |
| 200 | 5.793 | 5.679 |
| 300 | 5.783 | 5.693 |
| 400 | 5.761 | 5.683 |
| 500 | 5.806 | 5.702 |

**Supplementary Table 3.** The pressure sensitivities of the PSG resonator and the TCG resonator at different temperatures. The pressure range is from 0.001 kPa to 500 kPa.

| **Temperature** (℃) | PSG resonators *k*_1_ (kHz/kPa) | TCG resonators *k*_2_ (kHz/kPa) |
| --- | --- | --- |
| -40 | 24.137 | 0.187 |
| 0 | 24.134 | 0.185 |
| 40 | 24.113 | 0.190 |
| 80 | 24.141 | 0.221 |
| 120 | 24.163 | 0.195 |

# Supplementary References

[1] Wan, Z., Li, C., Liu, Y., Liu, Y. J., Xiao, X., & Han, S. Measuring optical reflectivity of graphene membranes using compensated Fabry-Perot interferometry. *Appl. Surf. Sci.* **639**, 8 (2023).

[2] Chen, Y. P. et al. Nano-optomechanical resonators for sensitive pressure sensing. *ACS Appl. Mater. Interfaces* **14**, 39211-39219 (2022).

[3] Tang, Z. Y., Fan, S. C., Xing, W. W., Guo, Z. S., & Zhang, Z. Y. An electrothermally excited dual beams silicon resonant pressure sensor with temperature compensation. *Microsyst. Technol.* **17**, 1481-1490 (2011).

[4] Liu, Y. J. et al. High-sensitivity graphene MOEMS resonant pressure sensor. *ACS Appl. Mater. Interfaces* **15**, 30479-30485 (2023).

[5] Chen. M. R. et al. An ultrahigh resolution pressure sensor based on percolative metal nanoparticle arrays. *Nat. Commun.* **10**, 9 (2019).

[6] Nair, R. R. et al. Fine structure constant defines visual transparency of graphene. *Science* **320**, 1308-1308 (2008).

[7] Dolleman, R. J., Davidovikj, D., Cartamil-Bueno, S. J., van der Zant, H. S. J. & Steeneken, P. G. Graphene squeeze-membrane pressure sensors. *Nano. Lett.* **16**, 568-571 (2016).

[8] Wan, Z. et al. Photothermal actuated miniature graphene resonator for high-sensitivity pressure detection. *IEEE Sens. J*. **23**, 22332-22339 (2023).

[9] Chen, C. Y. et al. Performance of monolayer graphene nanomechanical resonators with electrical readout. *Nat. Nanotechnol.* **4**, 861-867 (2009).
